# The Translational Paradox of Cancer Nanomedicine: Biological, Pharmacokinetic, and Manufacturing Barriers to Clinical Success

**DOI:** 10.3390/biology15171550

**Published:** 2026-09-04

**Authors:** Julia Jankowska, Łukasz Szeleszczuk, Dariusz Maciej Pisklak

**Affiliations:** Department of Organic and Physical Chemistry, Medical University of Warsaw, 1 Banacha Str., 02-097 Warsaw, Poland; s088868@student.wum.edu.pl (J.J.); dpisklak@wum.edu.pl (D.M.P.)

**Keywords:** cancer nanomedicine, clinical translation, nanocarriers, protein corona, enhanced permeability and retention effect, tumor microenvironment, pharmacokinetics, manufacturing scale-up, regulatory science

## Abstract

Cancer nanomedicine has generated extensive preclinical evidence of improved drug delivery, pharmacokinetics, and tolerability, yet its clinical impact has often remained modest. This narrative review examines the interconnected biological, pharmacokinetic, manufacturing, regulatory, and clinical factors underlying this translational paradox. A structured literature search was conducted primarily in PubMed and Google Scholar, focusing on studies published between 2022 and 2026 while retaining seminal earlier reports. Major biological barriers include protein corona formation, mononuclear phagocyte system clearance, heterogeneous enhanced permeability and retention, complex tumor microenvironments, and intratumoral heterogeneity. These factors limit circulation, tumor accumulation, tissue penetration, drug release, and interpatient reproducibility. Translation is further constrained by off-target accumulation, uncertain long-term toxicity, non-standardized experimental methods, batch-to-batch variability, scale-up challenges, and fragmented regulatory pathways. Clinical experience shows that successful products are dominated by relatively established platforms and reformulations of known anticancer agents, whereas many actively targeted or structurally complex systems have failed to demonstrate sufficient efficacy or safety. Future progress will require mechanism-driven design, human-relevant preclinical models, harmonized characterization, quality-by-design manufacturing, early regulatory integration, biomarker-guided patient selection, and adaptive clinical trials. Aligning nanoparticle engineering with biological and clinical realities is essential for achieving meaningful patient benefit.

## Simple Summary

Nanomedicine uses extremely small carriers to transport anticancer drugs through the body and, in principle, deliver them more selectively to tumors. Although many such systems perform very well in laboratory experiments and animal studies, only a limited number have produced clear and lasting benefits for patients. This review explains why this gap persists. After entering the bloodstream, nanoparticles can acquire a new biological coating, be removed by the immune system, accumulate in healthy organs, or fail to penetrate the complex and highly variable structure of human tumors. Their performance may also change during large-scale production, while differences among patients further reduce the predictability of treatment outcomes. The review also examines the clinical experience with approved, unsuccessful, and discontinued cancer nanomedicines. More reliable progress will require designs based on biological mechanisms, human-relevant experimental models, consistent manufacturing and testing standards, and clinical trials that select patients using measurable biological features indicating who is most likely to benefit. Addressing these challenges may help transform promising laboratory technologies into safer and more effective cancer treatments.

## 1. Introduction

### 1.1. What Is Nanomedicine?

Nanomedicine broadly refers to the application of nanoscale systems, typically ranging from 1 to 100 nm, in medical diagnosis, prevention, and therapy [1]. In oncology, nanocarriers are primarily developed to modify drug pharmacokinetics and biodistribution, improve solubility and stability, prolong systemic circulation, and reduce off-target exposure. These properties may improve treatment tolerability and, in selected settings, enhance drug delivery to tumor tissue [2].

### 1.2. Brief History of Nanomedicine

Modern nanomedicine emerged from advances in nanotechnology and drug-delivery science during the second half of the twentieth century [3,4,5,6]. A major clinical milestone was the 1995 approval of Doxil, the first liposomal formulation of doxorubicin, which demonstrated that nanoscale carriers could modify drug disposition and reduce systemic toxicity in a clinically meaningful manner [7,8]. This success stimulated the development of diverse nanocarrier platforms, including liposomes, polymeric nanoparticles, micelles, and dendrimers. In oncology, many subsequent platforms were designed to improve drug solubility, stability, pharmacokinetics, and tumor delivery, frequently exploiting passive accumulation mechanisms such as the enhanced permeability and retention (EPR) effect [9,10,11].

### 1.3. The Gap Between Preclinical Efficacy and Clinical Translation

Incorporating nanocarriers into cancer therapy has emerged as a prominent and extensively investigated area of oncological research. This innovative approach has demonstrated considerable potential for improving the safety and efficacy of pharmacotherapy through targeted drug delivery and reduced systemic toxicity. Preclinical studies conducted in murine models have yielded promising outcomes; however, the translation of these findings to clinical practice has produced variable and, in some cases, inconsistent results.

Despite the extensive body of literature highlighting the advantages of nanoscale delivery systems, relatively few formulations have received FDA or EMA approval. While numerous studies emphasize the advantageous properties of nanocarrier systems, including high drug-loading capacity and controlled drug release, considerably less attention is devoted to the limited clinical translation and high attrition rate of many proposed formulations. The translational paradox of cancer nanomedicine therefore warrants broader scientific discussion, particularly regarding the safety, efficacy, and clinical applicability of nanoformulation-based therapy in oncology.

Clinical failure of cancer nanomedicine is not caused by a single limitation, but by a cascade of interconnected biological, technological, manufacturing, and translational barriers.

Although several recent reviews have addressed specific aspects of nanomedicine translation, including the heterogeneity of the enhanced permeability and retention effect, nano–bio interactions, nanotoxicity, manufacturing challenges, and regulatory barriers, these issues are often discussed separately. The present review adopts a broader, integrated perspective by examining how biological and pharmacokinetic limitations interact with manufacturing reproducibility, standardization, regulatory requirements, and clinical trial outcomes. In addition, we directly compare clinically approved and sustained nanomedicines with formulations that failed during clinical development, were discontinued, or were withdrawn, thereby highlighting not only technological limitations but also the factors associated with translational success. By linking these different stages of the development pathway within a single framework, this review aims to provide a clinically oriented perspective on why promising preclinical nanomedicine platforms frequently fail to generate durable patient benefit and which strategies may be most relevant for improving future translation.

### 1.4. Literature Search and Review Approach

This article is an integrative narrative review rather than a systematic review or meta-analysis. It was informed by a structured literature search conducted primarily using PubMed/MEDLINE and Google Scholar. The search focused mainly on literature published between January 2022 and July 2026 in order to capture recent developments in cancer nanomedicine translation, while seminal and highly influential publications published before 2022 were retained when they provided essential mechanistic, historical, regulatory, or clinical context.

Searches were performed using combinations of terms related to cancer nanomedicine and its translational barriers. Representative Boolean combinations included (“cancer nanomedicine” OR nanoparticle* OR nanocarrier*) AND (“clinical translation” OR “translational barrier*” OR “clinical failure”); (“cancer nanomedicine” OR nanoparticle*) AND (“enhanced permeability and retention” OR EPR OR “tumor microenvironment” OR “protein corona” OR biodistribution OR pharmacokinetic*); and (“nanomedicine” OR nanoparticle*) AND (manufactur* OR scale-up OR “Good Manufacturing Practice” OR GMP OR regulatory OR commercialization OR “clinical trial*”). Additional targeted searches were performed for individual nanomedicine products, clinical trials, regulatory status, and specific biological or technological barriers discussed in the manuscript. The final literature search was performed in July 2026.

Eligible publications included original preclinical studies, clinical studies and trials, systematic reviews, meta-analyses, regulatory or authoritative clinical information, and review articles directly relevant to the biological, pharmacokinetic, toxicological, manufacturing, regulatory, or clinical translation of nanomedicines. Studies were prioritized when they provided quantitative data, mechanistic evidence, clinically relevant observations, or information concerning regulatory approval, clinical failure, withdrawal, or discontinuation. Publications were excluded when they were unrelated to biomedical nanomedicine, lacked sufficient relevance to the translational questions addressed in this review, duplicated information available from more authoritative sources, or provided insufficient methodological or clinical detail to support the corresponding discussion.

Search results were screened for relevance based on titles and abstracts, followed by evaluation of the full text when necessary to determine whether the publication contributed directly to the scope of the review. Reference lists of relevant reviews, meta-analyses, and landmark studies were also examined to identify additional publications. For clinical nanomedicine products and development programs, priority was given to peer-reviewed clinical evidence, trial registries, and regulatory information where available. Because the objective of the present work was an integrative narrative synthesis rather than an exhaustive systematic review or quantitative meta-analysis, study selection was guided by relevance to the predefined translational themes rather than by a formal systematic-review screening protocol.

Throughout the review, evidence derived from in vitro experiments, preclinical animal models, and human or clinical studies is distinguished wherever relevant. Quantitative findings are interpreted in the context of the experimental systems and methodological conditions from which they were derived, and observations obtained exclusively in preclinical models are not assumed to be directly representative of nanoparticle behavior in humans.

## 2. The Translational Paradox of Cancer Nanomedicine

The development of cancer nanomedicine is characterized by a persistent translational paradox, in which robust preclinical efficacy contrasts with limited and often modest clinical benefit. In mouse tumor models, nanocarrier-based drug delivery systems frequently demonstrate enhanced tumor accumulation, improved pharmacokinetics, and superior antitumor activity, largely attributed to optimized biodistribution and EPR effect [12,13]. These findings have generated considerable optimism regarding their therapeutic potential, particularly in improving the safety and efficacy of cytotoxic chemotherapy.

This preclinical success has been consistently observed across a wide range of experimental platforms, including multifunctional and stimuli-responsive nanocarriers designed to combine therapeutic modalities such as chemotherapy, immunotherapy, and ferroptosis induction [14,15]. Many of these systems demonstrate efficient tumor accumulation, significant tumor growth inhibition, and favorable in vivo toxicity profiles in murine models, further reinforcing the perceived potential of nanoscale drug delivery strategies [16]. Despite these encouraging experimental outcomes, comparable therapeutic efficacy has not been consistently reproduced in clinical settings, highlighting the substantial disconnect between preclinical nanomedicine performance and successful clinical translation [17].

Clinical evaluation of actively targeted immunoliposomal formulations has similarly yielded limited therapeutic benefit despite acceptable safety profiles, underscoring the broader challenges associated with translating mechanistically sophisticated nanocarrier systems into meaningful oncological outcomes [18]. In many cases, clinical improvements remain restricted to modest gains in progression-free survival or reduced systemic toxicity rather than substantial improvements in overall survival. Therefore, although nanomedicine platforms frequently demonstrate strong biological rationale and promising preclinical efficacy, relatively few formulations ultimately achieve durable clinical and regulatory success [19].

Understanding the biological, technological, and translational factors underlying this paradox remains a central challenge in the advancement of clinically effective cancer nanomedicine.

Among the different nanocarrier platforms, lipid nanoparticles (LNPs) represent an especially informative example of successful nanomedicine translation. Modern LNPs typically contain an ionizable lipid together with helper phospholipids, cholesterol, and a PEGylated lipid, enabling the encapsulation and protection of nucleic acid cargo while promoting cellular uptake and endosomal release. Importantly, the clinical validation of LNP technology has extended beyond oncology. The approval of the siRNA therapeutic patisiran provided an early demonstration that LNPs can enable systemic delivery of nucleic acids in humans, while the subsequent large-scale deployment of mRNA–LNP vaccines further established the feasibility of reproducible manufacturing, quality control, and clinical use of this platform. These developments have created a substantial technological and regulatory foundation that distinguishes LNPs from many less mature nanocarrier systems [20,21].

In cancer research, LNPs are being investigated particularly intensively as carriers for mRNA and siRNA. Preclinical studies have demonstrated that LNP-mediated delivery of immunomodulatory mRNAs can reshape the tumor microenvironment and induce systemic antitumor immunity. For example, LNP formulations delivering CD40 ligand- and CD40-encoding mRNAs, combined with dendritic-cell therapy, promoted tumor-specific T-cell responses, eradicated established tumors, inhibited distant lesions, and generated protection against tumor rechallenge in experimental models [22]. Other experimental approaches have used LNPs to deliver mRNAs encoding cytokines, tumor antigens, or mediators of immunogenic cell death, illustrating the versatility of the platform for cancer vaccination and local immune reprogramming. Nevertheless, these findings remain predominantly preclinical and therefore require cautious extrapolation to patients.

Clinical oncology data are now beginning to provide evidence that LNP-based RNA delivery may also contribute to meaningful therapeutic benefit. Individualized neoantigen therapy mRNA-4157 (V940; subsequently termed intismeran autogene), administered in combination with pembrolizumab after resection of high-risk melanoma, improved recurrence-free and distant metastasis-free survival compared with pembrolizumab alone in the randomized Phase IIb KEYNOTE-942 study, with sustained benefit observed during longer follow-up [23,24]. However, the clinical history of LNP-based oncology therapeutics also illustrates the limitations of the platform. For example, intravenous TKM-080301, an LNP formulation containing siRNA targeting polo-like kinase 1, showed an acceptable toxicity profile in patients with advanced hepatocellular carcinoma but did not demonstrate sufficient improvement in overall survival to support further development as a single agent [25]. Thus, the translational success of LNPs should not be attributed solely to their material composition. Their relative maturity reflects the convergence of efficient nucleic acid encapsulation and intracellular delivery, tunable ionizable lipid chemistry, modular formulation, scalable and reproducible manufacturing, and extensive accumulated development experience. At the same time, their application in oncology remains subject to many of the barriers discussed throughout this review, including biodistribution, immune interactions, tumor heterogeneity, intracellular delivery, patient selection, and the need to demonstrate clinical superiority rather than improved delivery alone.

## 3. Biological Barriers

### 3.1. Protein Corona

The formation of a protein corona on nanoparticle surfaces upon exposure to biological fluids is one of the major biological factors contributing to the translational limitations of cancer nanomedicine [26]. This corona consists of a dynamic layer of adsorbed serum proteins, traditionally classified into a rapidly exchanging “soft” corona and a more stable “hard” corona, with the latter contributing more strongly to the biological identity acquired by nanoparticles after administration. Importantly, this acquired identity may differ substantially from the physicochemical characteristics originally engineered during nanoparticle design, creating a major challenge for predicting nanoparticle behavior in vivo [27,28,29].

The formation of the protein corona can profoundly influence nanoparticle biodistribution by modifying surface properties and altering interactions with biological systems. The composition of the protein corona influences cellular uptake pathways, circulation half-life, tissue accumulation, and drug release kinetics. In particular, corona components may mask targeting ligands, enhance recognition by opsonins, and promote clearance by the mononuclear phagocyte system, thereby reducing tumor accumulation and limiting the efficiency of targeted delivery [30,31]. Therefore, nanoparticles that demonstrate favorable targeting properties under controlled experimental conditions may undergo substantial biological transformation after systemic administration, resulting in altered distribution profiles and reduced therapeutic exposure at the tumor site.

However, the translational relevance of protein corona-mediated effects is frequently underestimated in preclinical studies. Many experimental models rely on simplified biological environments, including standardized serum conditions and healthy animal models, while nanoparticle characterization is commonly performed before extensive biological interaction. Consequently, these systems fail to reproduce the patient-specific corona that rapidly forms after administration, leading to inaccurate prediction of nanoparticle pharmacokinetics and biodistribution [32].

Importantly, protein corona formation is not exclusively detrimental to nanoparticle targeting. Depending on its composition, the corona may preserve or even enhance selective interactions with target cells. Specific adsorbed proteins can function as endogenous targeting ligands, while deliberate preformation or engineering of the corona offers an opportunity to control the biological identity of nanoparticles before systemic administration. Experimental studies have demonstrated that albumin-rich coronas can enhance receptor-targeted nanoparticle association with cancer cells, whereas more complex serum-derived coronas may suppress the same targeting interaction [33]. More recently, biomimetic coronas containing selected transport and adhesion proteins have been engineered to reduce macrophage and complement recognition while retaining receptor-mediated targeting and improving tumor accumulation in vivo [34]. Thus, rather than being considered solely an unwanted consequence of exposure to biological fluids, the protein corona may represent a potentially programmable biological interface. Rational corona engineering could therefore complement conventional surface functionalization strategies, although its translational application will require reproducible control of corona composition and validation under physiologically and patient-relevant conditions.

Protein corona formation is a critical determinant of nanocarrier fate because it governs the biological identity acquired after systemic administration and thereby influences circulation persistence, organ distribution, tumor accumulation, and therapeutic efficacy. Improving translational predictability will require clinically relevant experimental systems, including patient-derived plasma models, humanized platforms, and systematic characterization of protein corona composition before clinical evaluation.

### 3.2. Mononuclear Phagocyte System (MPS)

**Figure 1:**
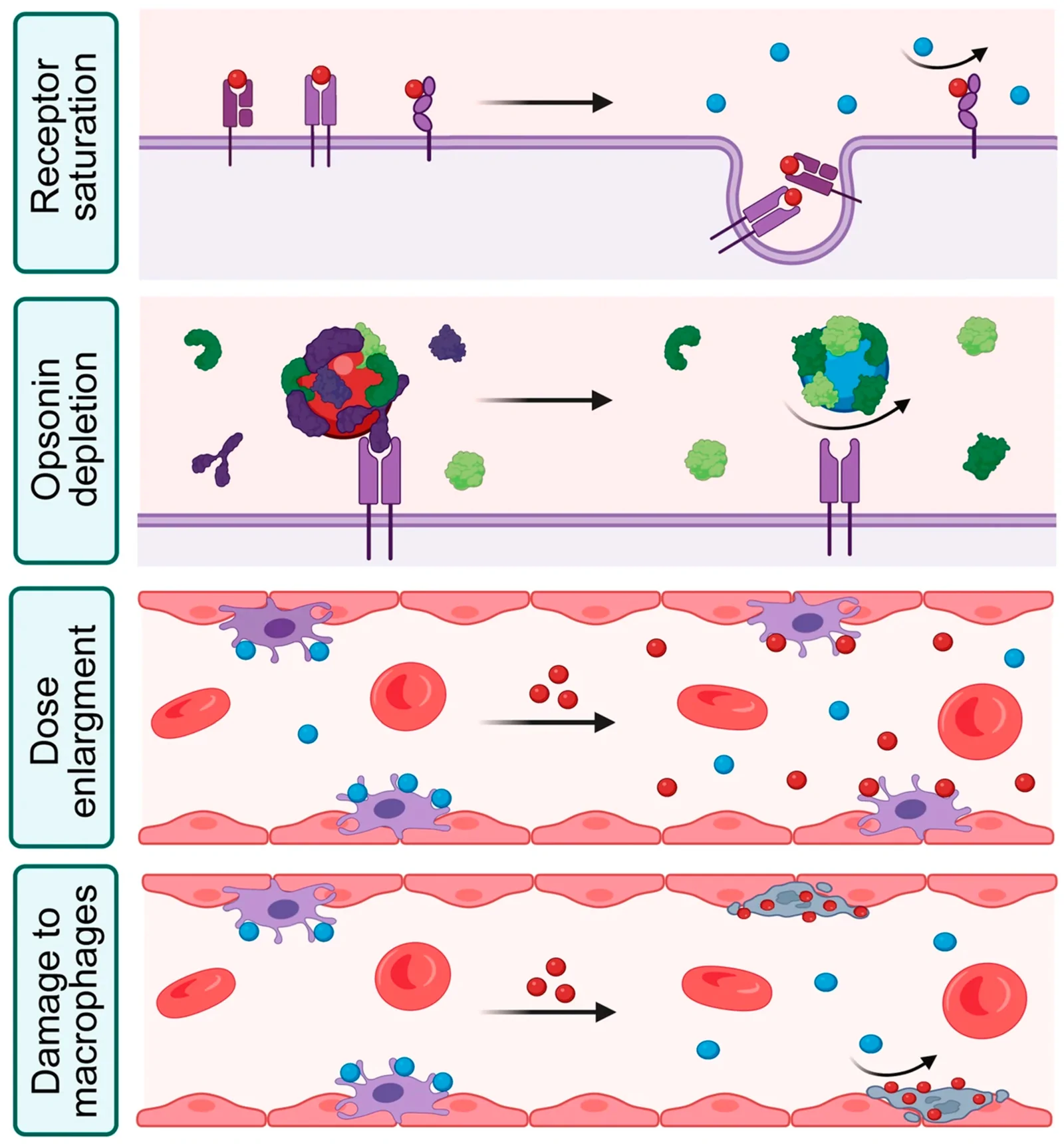
Different aspects of the combined mechanism of MPS blockade by nanoparticles. Reproduced from [29] under the Creative Commons Attribution 4.0 International license.

The primary mechanism underlying mononuclear phagocyte system (MPS)-mediated nanoparticle clearance is opsonization, whereby plasma proteins, including immunoglobulins, complement factors, and fibrinogen, promote recognition of nanoparticles by phagocytic cells. Opsonized nanoparticles are subsequently recognized by macrophages in the liver (Kupffer cells) and spleen through Fc receptors, complement receptors, and scavenger receptors, leading to rapid internalization and elimination from circulation. This immune-mediated clearance is a major determinant of nanoparticle biodistribution because shortened circulation time reduces the probability of tumor accumulation (Figure 1) [35,36,37].

A major translational challenge in nanomedicine is that the extent of immune-mediated clearance is frequently underestimated by preclinical models. Although rodent studies often demonstrate substantial tumor accumulation through enhanced permeability and retention (EPR)-mediated mechanisms, these effects are considerably less predictable in human tumors [38]. A frequently cited retrospective analysis of preclinical studies published between 2005 and 2015 estimated that a median of approximately 0.7% of the injected nanoparticle dose reached solid tumors [39]. However, this value should be interpreted cautiously rather than regarded as a universal benchmark of tumor delivery efficiency. The underlying studies were methodologically heterogeneous and differed substantially in nanoparticle composition and physicochemical properties, tumor models, dosing regimens, sampling schedules, and analytical methods. Moreover, the use of percent injected dose in the tumor (%ID) as a measure of delivery efficiency has subsequently been debated. Reanalysis of the underlying murine pharmacokinetic data using conventional exposure-based parameters demonstrated that the interpretation of nanoparticle tumor delivery can vary markedly depending on the pharmacokinetic metric selected [40]. Conversely, a later PBPK-based meta-analysis of 376 datasets from 200 tumor-bearing mouse studies reported a similar median tumor delivery efficiency of approximately 0.76% ID, but also demonstrated considerable dependence on sampling time, nanoparticle characteristics, and experimental conditions [41]. Thus, the approximately 0.7% value is best regarded as an approximate descriptor of the generally limited tumor accumulation observed across heterogeneous preclinical datasets rather than a fixed or universally representative property of cancer nanomedicines. Importantly, many preclinical systems rely on experimental conditions that do not fully reproduce human plasma composition, immune activation status, or interindividual variability in MPS function, limiting the predictive value of preclinical pharmacokinetic studies [42,43].

As discussed in Section 3.1, protein corona formation modulates MPS recognition by determining the extent of opsonization and subsequent macrophage uptake [43,44].

Beyond macrophage-mediated clearance, additional immune mechanisms further compromise nanoparticle performance. Complement activation following intravenous administration can promote nanoparticle elimination and, in some cases, induce hypersensitivity reactions, as reported for PEGylated liposomal formulations such as Doxil. Repeated administration introduces an additional challenge through the accelerated blood clearance (ABC) phenomenon, in which subsequent doses of PEGylated nanoparticles are rapidly removed due to anti-PEG antibody-mediated recognition. This issue is particularly relevant given increasing evidence of pre-existing anti-PEG antibodies in the general population resulting from widespread exposure to PEG-containing products. Moreover, variability in immune status, prior PEG exposure, and disease-associated immune alterations may lead to substantial differences in nanoparticle pharmacokinetics between patients [45].

MPS-mediated clearance remains one of the principal determinants of nanoparticle pharmacokinetics and biodistribution. Variability in immune function, complement activation, and anti-PEG immunity contributes to substantial interpatient differences in circulation time and therapeutic exposure. Improving clinical translation will therefore require immune-relevant experimental models capable of predicting patient-specific clearance mechanisms.

### 3.3. Limitations and Translational Relevance of the EPR Effect

The enhanced permeability and retention (EPR) effect has historically provided an important mechanistic basis for passive nanoparticle accumulation in solid tumors. In many preclinical tumor models, particularly rapidly growing murine xenografts, abnormal and highly permeable tumor vasculature combined with impaired lymphatic drainage can facilitate the extravasation and retention of nanoscale drug carriers. These observations have contributed substantially to the development of cancer nanomedicine. However, the magnitude of EPR-mediated accumulation varies considerably among experimental models and depends on tumor type, vascular architecture, perfusion, nanoparticle characteristics, and the time point at which accumulation is assessed [46,47,48].

Evidence from human tumors indicates a substantially more heterogeneous situation. The EPR effect should therefore not be considered either universally present or entirely absent in patients. Human tumors differ markedly between cancer types, individual patients, and even separate lesions within the same patient with respect to vascular density, vessel permeability, perfusion, extracellular matrix composition, interstitial fluid pressure, stromal content, and lymphatic function. Consequently, nanoparticle accumulation may be substantial in selected tumors but limited in others. This variability reduces the reliability of EPR-mediated delivery as a universal strategy for clinical tumor targeting.

An important source of the preclinical–clinical discrepancy is the limited ability of conventional animal models to reproduce the structural and physiological complexity of human tumors. Rapidly growing subcutaneous xenografts may display greater vascular permeability and less developed stromal barriers than many spontaneously developing human malignancies. Differences in tumor growth rate, vascular organization, immune microenvironment, extracellular matrix density, and nanoparticle clearance between species further complicate direct extrapolation. Accordingly, successful EPR-mediated accumulation in a murine tumor model should not by itself be interpreted as evidence that comparable delivery will occur in patients.

**Figure 2:**
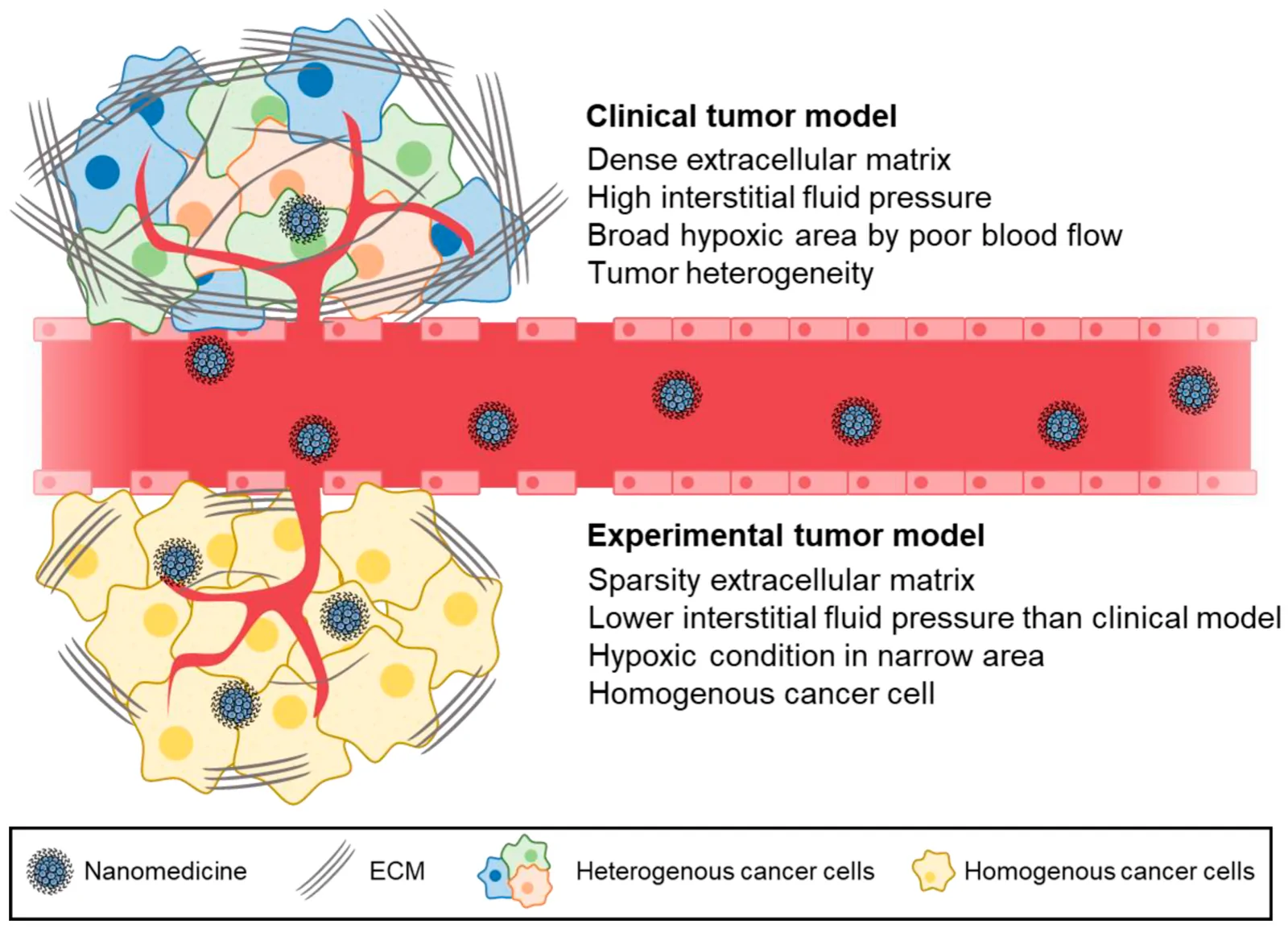
Schematic comparison of conventional experimental tumor models and human tumors. Relative to many rapidly growing xenograft models, human tumors may exhibit denser extracellular matrices, higher and more heterogeneous interstitial fluid pressure, broader hypoxic regions, greater cellular heterogeneity, and more variable vascular architecture, all of which can influence nanoparticle extravasation, penetration, and EPR-mediated accumulation. Reproduced from [48] under the Creative Commons Attribution 4.0 International license.

The translational significance of the EPR effect is therefore better viewed as context-dependent rather than as a universal property of solid tumors. Future clinical development may benefit from identifying tumors and patients in whom vascular and microenvironmental characteristics are favorable for nanoparticle accumulation rather than assuming comparable passive targeting across unselected populations. Furthermore, active targeting should not be regarded as a general solution to limited EPR-mediated delivery, because receptor-specific ligands do not necessarily increase the amount of nanocarrier that extravasates from the circulation into tumor tissue. Their principal advantage may instead occur after tumor access, by enhancing recognition, binding, or internalization by selected target cells. Thus, tumor accumulation, intratumoral penetration, and cellular uptake should be considered distinct and sequential barriers to effective nanomedicine delivery (Figure 2).

### 3.4. Tumor Microenvironment

**Figure 3:**
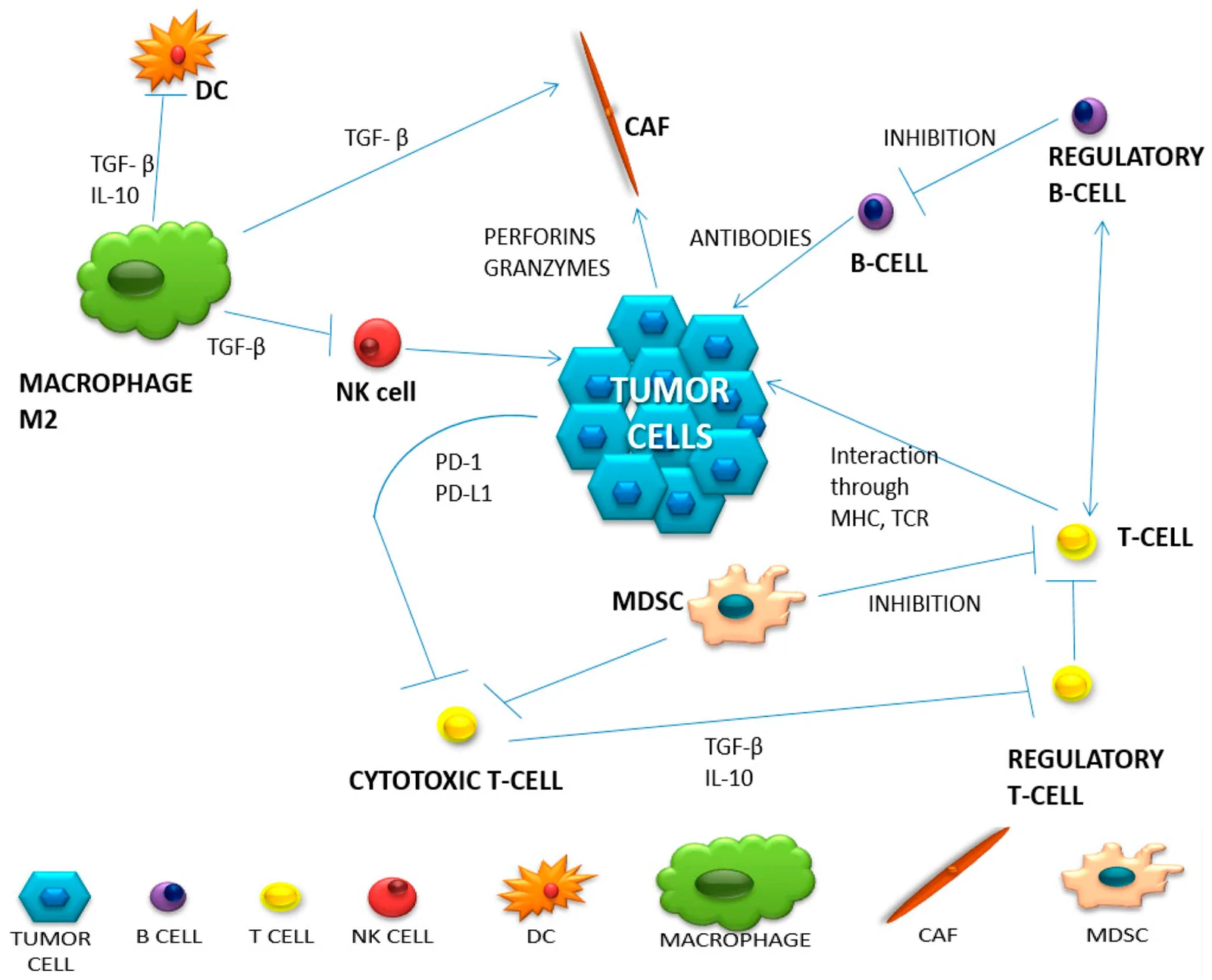
The network of intercellular interactions in the tumor microenvironment. Reproduced from [38] under the Creative Commons Attribution 4.0 International license.

The tumor microenvironment (TME) is a dynamic system comprising tumor cells, immune cells, and non-cellular components, including the extracellular matrix, cytokines, interleukins, and extracellular vesicles. Interactions among these components regulate tumor development, progression, and communication with surrounding tissues. Tumor heterogeneity and continuous molecular and phenotypic changes influence the tumor immunophenotype, promoting immune evasion, reducing therapeutic efficacy, and contributing to treatment resistance (Figure 3) [49,50].

The TME plays a central role in cancer progression by promoting proliferation, angiogenesis, immune evasion, metastasis, and therapy resistance. During tumor development, it shifts from a tumor-suppressive to a tumor-promoting environment, where cancer cells reprogram stromal and immune cells to support their survival. Tumor-associated macrophages and neutrophils secrete growth-promoting factors, cancer-associated fibroblasts remodel the extracellular matrix, and pro-angiogenic mediators such as vascular endothelial growth factor (VEGF) stimulate angiogenesis [51]. In parallel, the TME promotes immunosuppression through PD-1/PD-L1 signaling, regulatory immune cells, and metabolic alterations that impair cytotoxic T-cell activity, while facilitating metastasis through epithelial–mesenchymal transition, extracellular matrix remodeling, and cancer cell migration. Together, these processes promote resistance to chemotherapy, radiotherapy, anti-angiogenic therapy, and immunotherapy [52,53,54].

The TME is increasingly recognized as an important therapeutic target in oncology because its composition and molecular characteristics can serve as prognostic and predictive biomarkers. A better understanding of TME interactions may improve personalized treatment strategies, optimize first-line therapy selection, enhance the efficacy of conventional anticancer treatments, and help overcome treatment resistance [54,55].

From the perspective of nanomedicine, the TME primarily determines whether systemically circulating nanocarriers can access and penetrate tumor tissue. Abnormal vascular architecture, elevated interstitial fluid pressure, dense extracellular matrix, and stromal barriers can sequentially restrict extravasation and subsequent movement through the tumor interstitium. These features therefore influence the amount and spatial distribution of nanocarriers that reaches different tumor regions. Because conventional preclinical models often reproduce these transport barriers incompletely, their predictive value for human tumor delivery remains limited.

### 3.5. Intratumoral Heterogeneity

Intratumoral heterogeneity (ITH) describes the coexistence of genetically, phenotypically, and functionally distinct cancer cell subpopulations within a single tumor, reflecting its dynamic and complex nature [56]. ITH arises primarily from genomic instability and clonal evolution, while epigenetic alterations, transcriptional plasticity, and interactions with the tumor microenvironment further increase genetic, phenotypic, and metabolic diversity [57,58].

ITH enables tumors to adapt under selective pressure, promoting disease progression and therapeutic resistance. Spatial and temporal heterogeneity within the tumor microenvironment favors the expansion of subclones with survival advantages. Distinct driver alterations, including *EGFR*, *KRAS*, and *MET*, may therefore emerge or coexist in different tumor regions [59].

ITH also contributes to metastasis through tumor-derived exosomes, which promote premetastatic niche formation, and through heterogeneous activation of signaling pathways, including PI3K/AKT/mTOR and MEK/ERK, that support metabolic reprogramming and resistance to anoikis [59,60].

Immune heterogeneity further facilitates immune escape through regional differences in immune cell infiltration, progressive T-cell exhaustion, and variable expression of immune checkpoints such as PD-L1. ITH also promotes adaptation to hypoxia, nutrient deprivation, and oxidative stress through the formation of distinct selective niches and heterogeneous activation of stress-response pathways [57,61,62].

A major clinical consequence of ITH is therapeutic resistance. Tumor subclones differ in treatment sensitivity, allowing resistant populations to survive and drive recurrence. Therapeutic interventions may further increase intratumoral heterogeneity, highlighting the need for rational combination therapies targeting multiple tumor subpopulations simultaneously [63,64].

From the perspective of nanomedicine, ITH becomes particularly important after a nanocarrier has reached the tumor tissue. Spatial differences in cellular phenotype, target-receptor expression, metabolic state, proliferative activity, and treatment sensitivity can result in unequal cellular recognition, internalization, drug exposure, and therapeutic response across different tumor regions. Consequently, even when adequate tumor-level accumulation is achieved, the delivered nanomedicine may not reach or effectively treat all malignant subpopulations. ITH should therefore be considered primarily as a determinant of heterogeneous cellular exposure and response rather than as another description of the physical transport barriers discussed in Section 3.4.

Taken together, the biological barriers discussed in Section 3.1, Section 3.2, Section 3.3, Section 3.4 and Section 3.5 form a sequential cascade: acquisition of a biological identity influences immune recognition and systemic clearance; limited circulating exposure constrains tumor access; the EPR effect and TME determine extravasation and tissue penetration; and ITH ultimately contributes to heterogeneous cellular exposure and therapeutic response.

## 4. Technology–Biology Interactions, Pharmacokinetics, and Safety Limitations

### 4.1. Pharmacokinetics

**Figure 4:**
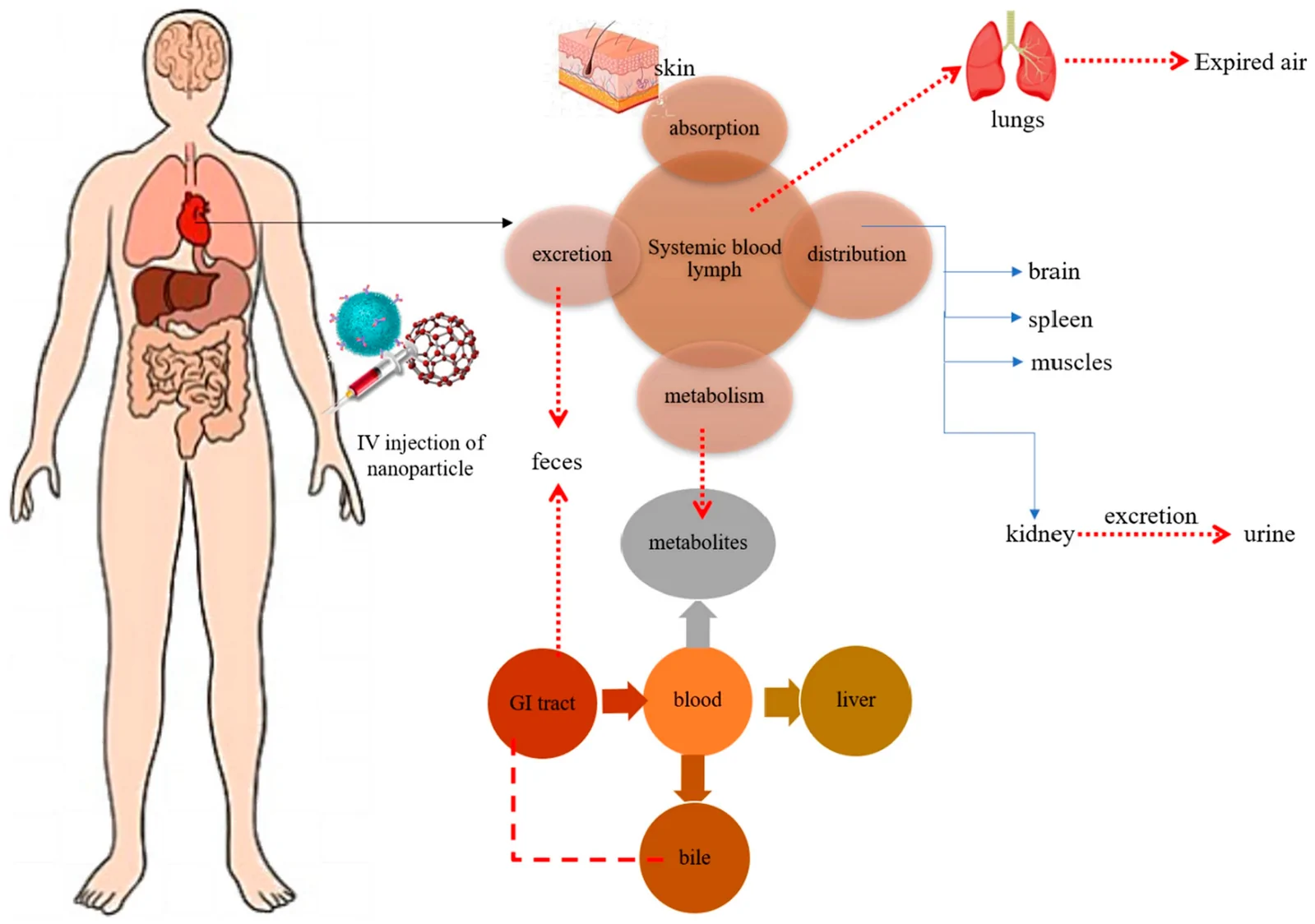
Overall ADME mechanism and fate of nanoparticles. Reproduced from [38] under the Creative Commons Attribution 4.0 International license.

The pharmacokinetics of nanomedicine encompasses the absorption, distribution, metabolism, and excretion (ADME) of nanoparticle-based drug delivery systems, including the behavior of the nanocarrier itself, the fate of the carrier material, and the release and disposition of the encapsulated or associated drug. Unlike conventional pharmaceuticals, nanoparticles exhibit distinct pharmacokinetic properties influenced by physicochemical characteristics such as size, shape, surface charge, and coating composition (Figure 4) [65,66].

Nanoparticles are primarily administered through oral and intravenous routes. Intravenous administration enables direct entry into systemic circulation, bypassing absorption, whereas orally administered nanoparticles must overcome biological barriers, including gastric pH variations, intestinal enzymes, and the gastrointestinal mucus layer [65]. Their absorption may be facilitated by improved dissolution, mucoadhesion, and transcellular uptake through endocytosis [67].

Following systemic administration, nanoparticle pharmacokinetics is strongly influenced by interactions with plasma proteins and the mononuclear phagocyte system, as discussed in Section 3.1 and Section 3.2. These interactions can alter circulation time, biodistribution, and clearance and therefore represent an important link between the biological identity of a nanocarrier and its systemic pharmacokinetic behavior.

Nanoparticle biodistribution is influenced by multiple physicochemical factors, particularly size, surface properties, shape, and formulation characteristics. Although size-dependent trends have been described, reported ranges should be considered approximate and dependent on nanoparticle platform, flexibility, surface coating, and aggregation state. Smaller nanoparticles may undergo more efficient renal elimination, whereas larger or less deformable particles are more susceptible to uptake by phagocytic cells and accumulation in organs such as the liver and spleen. Although size optimization has traditionally been used to maximize circulation and EPR-mediated accumulation, this relationship is highly context-dependent and poorly predictive in human tumors [65]. However, the extent of tumor accumulation remains highly variable and depends on tumor biology and the properties of the nanocarrier. Surface charge also influences biodistribution, with neutral nanoparticles generally exhibiting longer circulation times than positively charged formulations, which are more rapidly cleared [68,69,70].

Surface modification with polyethylene glycol (PEG), known as PEGylation, can reduce opsonization and recognition by the MPS, thereby increasing circulation half-life [68]. For example, PEGylated liposomal doxorubicin (Doxil) achieves substantially higher plasma concentrations and reduced clearance compared with free doxorubicin [67]. Similarly, neutral Ferumoxtran-10 nanoparticles exhibit prolonged blood half-lives compared with non-PEGylated formulations [68].

The metabolism and degradation of nanomedicines depend primarily on the composition of the carrier material rather than following the metabolic pathways of conventional small-molecule drugs. Biodegradable nanoparticles composed of materials such as polylactic acid (PLA), poly(lactic-co-glycolic acid) (PLGA), and lipids undergo enzymatic and chemical degradation, generating products that are subsequently eliminated through renal or hepatobiliary pathways. In contrast, non-biodegradable nanoparticles, including gold nanoparticles, may exhibit prolonged retention and require extended periods for clearance. The fate of the carrier material must be distinguished from the pharmacokinetics of the released active drug, which may follow different absorption, distribution, metabolism, and elimination pathways [65,71].

Drug release from nanocarriers represents a critical determinant of therapeutic activity. The concentration of total drug measured within tumor tissue does not necessarily reflect the amount of free, pharmacologically active drug available at the relevant tumor compartment. Substantial accumulation of nanomedicines within tumor tissue does not necessarily reflect adequate intracellular concentrations of the released, pharmacologically active drug at the target site. Thus, nanoparticle accumulation alone does not guarantee effective drug delivery or therapeutic response [72,73].

Excretion of nanomedicines may occur through renal and/or hepatobiliary pathways, depending on nanoparticle size, composition, biodegradability, and physicochemical properties. Smaller nanoparticles may undergo renal filtration, whereas larger particles are typically processed through hepatobiliary elimination. Efficient clearance is important for minimizing prolonged retention and potential toxicity [1].

The pharmacokinetics of nanomedicines is considerably more complex than that of conventional small-molecule drugs and requires specialized analytical techniques and physiologically based pharmacokinetic (PBPK) modeling to accurately characterize nanocarrier behavior and drug release in vivo [66]. Major pharmacokinetic advantages of nanomedicines include prolonged circulation time, improved bioavailability, enhanced tissue targeting, and reduced systemic toxicity compared with free drug formulations [56]. Nevertheless, challenges remain, including incomplete understanding of nano-bio interactions, difficulties in predicting biodistribution across species, and the need for standardized pharmacokinetic evaluation methods [66,68].

Overall, the interplay between nanoparticle properties, biological interactions, carrier degradation, and drug release determines nanomedicine pharmacokinetics. Therefore, each nanoparticle formulation requires individual characterization, as generalized principles cannot reliably predict the behavior of all nanomaterial platforms [65,68].

### 4.2. The Technology–Biology Interface in Cancer Nanomedicine

The clinical performance of a nanocarrier is determined not only by its engineered physicochemical properties but by how these properties interact with the biological characteristics of the tumor and the host. Particle size, morphology, surface chemistry, charge, deformability, carrier composition, targeting ligands, and drug-release kinetics may influence circulation, vascular extravasation, tissue penetration, cellular uptake, and intracellular drug availability. However, the effect of each design parameter is highly context-dependent because tumors differ markedly in vascular permeability, perfusion, stromal organization, extracellular matrix density, interstitial fluid pressure, immune-cell composition, and receptor expression. Consequently, a nanocarrier optimized for one biological environment may perform poorly in another, even when its physicochemical characteristics are highly reproducible.

Different nanocarrier technologies may therefore be advantageous at different stages of the delivery process. Long-circulating liposomal and PEGylated systems can reduce rapid systemic clearance and prolong drug exposure, but prolonged circulation does not necessarily translate into efficient tumor penetration. Small or deformable nanocarriers may penetrate tissue more effectively in selected tumors, whereas larger systems may achieve prolonged retention but remain confined near tumor vasculature. Similarly, receptor-targeted nanocarriers can enhance cellular recognition and internalization when the corresponding target is sufficiently expressed and accessible, but they cannot compensate for poor vascular extravasation or dense stromal barriers. Thus, targeting ligand density, particle dimensions, surface chemistry, and release kinetics should be selected in relation to the dominant biological barrier rather than optimized independently of tumor biology.

Carrier composition and drug-release behavior further illustrate this technology–biology interdependence. Liposomes, polymeric nanoparticles, lipid nanoparticles, micelles, and inorganic systems differ in stability, degradation pathways, intracellular trafficking, and mechanisms of cargo release. For nucleic acid delivery, for example, efficient cellular uptake must be followed by endosomal escape and cytosolic delivery, whereas conventional cytotoxic drugs may require controlled extracellular or intracellular release to achieve effective local exposure. At the same time, hypoxia, acidic pH, enzymatic activity, oxidative stress, and other features of the tumor microenvironment can potentially be exploited by stimuli-responsive systems, although the spatial and interpatient heterogeneity of these conditions may limit reproducibility. The most clinically relevant design strategy may therefore be one in which nanocarrier properties are matched prospectively to the specific biological barriers, target expression profile, and microenvironmental characteristics of the intended tumor type.

### 4.3. Off-Target Accumulation and Long-Term Toxicity

Nanomedicine continues to face significant translational barriers that limit its clinical efficacy and safety. As discussed in Section 3.2, quantitative estimates of nanoparticle tumor accumulation vary substantially according to nanoparticle platform, tumor model, pharmacokinetic metric, sampling time, and analytical methodology. Nevertheless, the available preclinical evidence consistently indicates that systemic nanoparticle delivery to solid tumors is highly variable and frequently limited. This variability, together with premature drug release and nonspecific biodistribution, may contribute to off-target exposure and toxicity in healthy tissues. Consequently, increasing attention has been directed toward active targeting and transcytosis-mediated delivery strategies to improve tumor selectivity and therapeutic efficacy [12]. As discussed in Section 3.3, the clinical contribution of the EPR effect is highly heterogeneous, and neither passive nor active targeting alone can overcome the sequential barriers of systemic circulation, tumor extravasation, intratumoral penetration, and cellular uptake [2].

Importantly, safety considerations in cancer nanomedicine should distinguish adverse effects associated with therapeutic nanoformulations from toxicological effects reported for engineered nanomaterials more broadly. Much of the available evidence regarding reproductive and developmental toxicity originates from preclinical nanotoxicology studies of materials such as silica, silver, gold, titanium dioxide, nickel-based nanoparticles, and carbon nanotubes rather than from clinical studies of therapeutic cancer nanomedicines. Experimental studies have reported reproductive-organ accumulation, altered steroidogenesis, oxidative stress, mitochondrial dysfunction, inflammatory responses, and tissue injury following exposure to some of these materials [74,75,76,77]. However, these findings were obtained under heterogeneous experimental conditions that differ substantially in material composition, dose, route of administration, exposure duration, and intended biomedical use. They are therefore relevant for identifying potential toxicological mechanisms and safety signals but should not be directly extrapolated to clinically administered oncology nanoformulations. Accordingly, evaluation of long-term nanomedicine safety should remain formulation-specific and should consider the physicochemical properties, biodistribution, degradation, persistence, and exposure profile of the individual therapeutic platform.

## 5. Manufacturing and Regulatory Bottlenecks

### 5.1. Challenges in Large-Scale, Cost-Effective Manufacturing

The large-scale manufacturing of multifunctional nanocarriers remains a major obstacle to the clinical translation and commercialization of nanomedicine platforms. In contrast to conventional pharmaceutical formulations, nanoparticle-based systems require stringent control over multiple physicochemical attributes, including particle size distribution, morphology, surface charge, colloidal stability, and drug-loading efficiency, all of which critically influence biodistribution, therapeutic efficacy, and safety profiles [78,79,80]. Preserving these critical quality attributes during scale-up is particularly challenging, as even minor deviations in synthesis conditions may produce substantial batch-to-batch variability and compromise biological performance [81]. Although noncovalent self-assembly and co-assembly strategies have emerged as promising manufacturing approaches due to their relative simplicity, lower production costs, and improved reproducibility, their translation to industrial-scale production still necessitates extensive process optimization and validation under Good Manufacturing Practice (GMP) conditions [82,83].

A major limitation in current nanoparticle manufacturing is the absence of harmonized and standardized production protocols across laboratories and industrial facilities. Variability in key synthesis parameters, including precursor concentration, solvent composition, pH, temperature, reaction time, and mixing kinetics, frequently results in poor inter-batch and inter-laboratory reproducibility. In addition, inconsistencies in purification procedures, storage conditions, and analytical characterization methodologies further complicate reliable comparison between studies and impede regulatory assessment [84]. Such variability directly affects nanoparticle physicochemical properties, including hydrodynamic diameter, polydispersity index, zeta potential, surface functionalization, and encapsulation efficiency, thereby limiting manufacturing robustness and translational reliability [85]. Consequently, the implementation of standardized operating procedures, validated characterization platforms, and rigorous quality-control frameworks is increasingly recognized as essential for reproducible nanoparticle production and regulatory compliance [86].

The large-scale, cost-effective production of metal nanoparticles presents additional multifactorial challenges. Precise control over nanoparticle size, morphology, and surface characteristics is essential because these parameters directly influence optical, catalytic, and biomedical performance. However, many conventional synthetic approaches rely on high-temperature processing or chemically aggressive reaction environments that are difficult to reproduce consistently under industrial-scale conditions. Furthermore, traditional synthesis methods frequently employ toxic reducing agents and generate hazardous by-products, raising significant safety, environmental, and regulatory concerns that further limit scalability. The environmental impact of metal nanoparticles also represents a critical consideration, particularly given the documented ecotoxicity of silver and other metallic nanomaterials in aquatic and biological systems. Consequently, comprehensive toxicological assessment and the development of environmentally sustainable synthesis strategies are increasingly recognized as prerequisites for responsible commercialization. In addition, economic constraints, including high energy demands, raw-material costs, process optimization requirements, and the need for continuous large-scale production, remain substantial barriers to industrial implementation. Collectively, these challenges underscore the necessity for scalable green-manufacturing technologies, robust and standardized characterization methodologies, GMP-compliant production workflows, and comprehensive risk-assessment frameworks to enable the reliable and economically sustainable commercialization of nanoparticle-based systems [87,88].

### 5.2. Poor Standardization Across Studies and Evaluation Methods

The successful clinical advancement of nanomedicine is further complicated by persistent methodological and translational limitations. The absence of standardized frameworks for nanoparticle characterization, combined with significant variability in particle size, morphology, and surface charge, complicates the reproducibility and comparability of experimental findings. These challenges, together with stringent regulatory requirements, contribute to the relatively low number of nanomedicine formulations that successfully achieve clinical approval [89].

Furthermore, a persistent challenge in nanomedicine is the poor standardization of experimental protocols and evaluation methods. At the preclinical level, key parameters such as cell culture media, serum supplementation, incubation conditions, and nanoparticle dosimetry are often applied without consensus, making it difficult to obtain consistent and reproducible results. The lack of standardized sample preparation procedures, variability in cell and animal models, and the need for specialized bioinformatics expertise further complicate nanotoxicity assessment and hinder meaningful comparisons across studies [1,90].

Insufficient standardization across polymeric nanoparticle (PNP) studies further impedes reliable cross-study comparison and clinical translation. Preclinical investigations frequently employ heterogeneous physicochemical characterization techniques, drug-loading methodologies, and release-kinetics protocols, thereby limiting the assessment of batch-to-batch reproducibility and often generating inconsistent or contradictory efficacy outcomes. Moreover, the enhanced permeability and retention (EPR) effect, widely used as a metric of passive tumor targeting, exhibits pronounced heterogeneity across tumor models and is quantified using nonuniform approaches to vascular permeability and nanoparticle accumulation [91]. This lack of methodological consistency reduces the predictive validity of preclinical data. Safety evaluation also remains insufficiently standardized. Toxicological assessments vary considerably with respect to cell-line selection, dosing regimens, exposure duration, and measured endpoints, thereby hindering systematic risk assessment and the establishment of robust safety profiles for PNP-based therapeutics [86].

## 6. Clinical Reality

In contrast to the predominantly mechanistic and preclinical evidence discussed in the preceding sections, this section focuses specifically on nanomedicine formulations that have entered clinical development or received regulatory authorization, thereby allowing the translational relevance of experimentally identified barriers to be assessed against actual clinical outcomes.

The analysis of clinically approved and late-stage nanomedicine reveals several important trends in the development and clinical translation of nanocarrier-based cancer therapeutics. Based on the data summarized in Table 1 and Table 2, liposomal formulations represent the predominant class among FDA-approved nanomedicines used in oncology, including both currently established products such as Doxil and Vyxeos and historically approved formulations such as DaunoXome and Marqibo. This predominance may be attributed to their favorable safety profiles, highlighting the critical importance of biocompatibility, physicochemical stability, and clinical feasibility in successful translational outcomes. However, regulatory approval and subsequent market availability represent distinct aspects of clinical translation, and discontinuation of an approved product should not automatically be interpreted as evidence of inadequate efficacy or unacceptable toxicity. The reasons for loss of market availability differ substantially among individual formulations. DaunoXome was previously approved for advanced HIV-associated Kaposi sarcoma and was subsequently commercially discontinued, whereas the available regulatory documentation does not establish its discontinuation as a consequence of pharmacological failure. DepoCyt/DepoCyte provides a particularly clear example of commercially driven discontinuation: its European marketing authorization was withdrawn at the request of the marketing authorization holder following a decision to permanently discontinue marketing for commercial reasons. Marqibo represents a different regulatory pathway; it received accelerated FDA approval in 2012, and approval of its New Drug Application was subsequently withdrawn in 2022 following a voluntary request from the sponsor. These examples demonstrate that commercial discontinuation, regulatory withdrawal, and clinical development failure should be treated as distinct translational outcomes rather than grouped under a single category of nanomedicine failure.

In contrast, more structurally complex nanocarrier systems, such as dendrimers and inorganic nanoparticles, remain underrepresented in clinical oncology despite encouraging preclinical performance. This disparity reflects the broader challenges associated with the clinical translation of nanomedicine-based platforms in cancer therapy. Although advanced nanocarriers have been designed to achieve active targeting, controlled drug release, and improved tumor specificity, many have failed to demonstrate sufficient clinical benefit. The reasons for unsuccessful or incomplete clinical translation vary substantially among individual platforms and should not be treated as equivalent. MM-302 was terminated after a futility analysis indicated insufficient clinical benefit, whereas development of MM-310 was discontinued because emerging safety data suggested an unfavorable therapeutic index. The history of BIND-014 was more complex, involving indication-specific development decisions, enrollment and strategic considerations, and subsequent financial restructuring of the sponsor. Similarly, discontinuation of previously approved products should not automatically be interpreted as pharmacological failure, as market withdrawal may reflect commercial or regulatory considerations rather than inadequate efficacy or unacceptable toxicity. These distinctions are summarized in Table 2. Among nucleic acid-based nanomedicines, MRX34 provides a clear example of safety-limited development, as its Phase I study was terminated following serious immune-related adverse events. CALAA-01 also did not progress beyond early clinical development; however, the primary trial registry does not specify a single definitive reason for termination [111,113,115,116].

Another key observation is that the majority of approved nanomedicines are based on reformulations of established chemotherapeutic agents rather than novel molecular entities. Frequently used active compounds include doxorubicin, paclitaxel, cisplatin derivatives, and daunorubicin. This trend suggests that the primary clinical advantage of nanocarrier systems lies in the optimization of pharmacokinetic properties, reduction in systemic toxicity, and improvement of drug tolerability rather than the introduction of new mechanisms of action. However, improved pharmacokinetic behavior alone has often been insufficient to achieve clinical superiority. Several formulations, including SPI-077, CRLX101, and NK105, demonstrated improved drug delivery characteristics but failed to provide meaningful improvements in therapeutic efficacy, emphasizing the importance of demonstrating clear clinical advantages over conventional therapies [119,124,127].

ThermoDox illustrates how promising preclinical and early clinical findings may nevertheless fail to translate into regulatory approval, as its Phase III development program was discontinued following a futility assessment. Other investigational nanoformulations remain under clinical evaluation, illustrating that lack of approval may reflect fundamentally different translational outcomes [128]. The withdrawal or discontinuation of multiple nanoformulations indicates that the main obstacles in nanomedicine development are not limited to nanoparticle design but also involve complex interactions between nanocarriers, tumor biology, immune responses, and clinical treatment environments. Overall, these trends highlight a substantial gap between the diversity of nanocarrier systems developed in preclinical research and the limited subset that successfully achieves and maintains clinical translation, demonstrating that future advances in cancer nanomedicine will require not only improved delivery technologies but also stronger clinical validation and a clearer demonstration of patient benefit.

## 7. Strategies to Overcome Translational Failure: From Established Approaches to Emerging Concepts

The strategies discussed in this section differ substantially in their level of technological and clinical maturity. Some, including standardized physicochemical characterization, GMP-compliant manufacturing, Quality by Design (QbD), Process Analytical Technology (PAT), and early regulatory integration, are already established components of pharmaceutical development and manufacturing. Other approaches, such as human-relevant preclinical models, mechanism-driven nanomedicine design, biomarker-guided patient selection, and nanomedicine-specific adaptive clinical trial approaches, are emerging and increasingly supported by experimental or clinical evidence but are not yet routinely implemented across cancer nanomedicine development. Finally, strategies including deliberate protein-corona engineering and AI-assisted patient stratification remain largely conceptual or future-oriented in this context. The relative maturity of these approaches is summarized in Table 3.

### 7.1. Mechanism-Driven Nanomedicine Design

The translational gap in nanomedicine remains substantial. Across the broader nanomedicine field, approximately 10,000 publications are produced annually, whereas only around 200 organic and inorganic nanomedicines have progressed to clinical evaluation [129]. These figures are not specific to cancer nanomedicine but illustrate the broader disparity between experimental activity and clinical translation. This disparity reflects a persistent inability to predict and control nanoparticle behavior once materials encounter complex biological environments [129]. Mechanism-driven nanomedicine design seeks to overcome this limitation by replacing empirical, materials-first development with a framework that explicitly incorporates biotransformation and other nano–bio interactions into nanoparticle design. Rather than treating biological transformations as secondary phenomena, this approach considers them central determinants of therapeutic performance [130].

As discussed by Neuer et al., clinical translation remains limited because nanomaterial chemistry is insufficiently understood and controlled throughout the nanoparticle lifecycle, from storage and circulation to metabolism and excretion. Although these processes have been extensively investigated for organic nanomaterials such as liposomes and micelles, comparable knowledge of the physical and chemical transformation of inorganic nanomedicines in vivo remains limited [129]. These transformations alter nanoparticle composition, structure, and surface chemistry, thereby influencing biodistribution, efficacy, and safety. Consequently, nanoparticles frequently behave differently in vivo than predicted from their initial physicochemical properties. This knowledge gap is further compounded by the poor correlation between in vitro and in vivo findings, as highlighted by Stater et al., indicating that current preclinical screening strategies often fail to reliably predict biological performance [131,132,133].

As discussed in Section 3.1, the biological identity acquired by nanoparticles after exposure to biological fluids can substantially modify their interactions with cells and tissues. Mechanism-driven design therefore requires these nano–bio interactions to be considered prospectively during nanoparticle development rather than evaluated only after formulation optimization.

An additional consideration highlighted by Stater et al. is the occurrence of ancillary effects, defined as payload-independent biological responses elicited by the nanoparticle material itself. These responses range from subtle modulation of cell signaling, gene expression, and differentiation to oxidative stress, complement activation, endothelial dysfunction, and cytotoxicity. Although such effects may contribute to adverse outcomes, they can also be therapeutically advantageous, for example, by enhancing vaccine immunogenicity or sensitizing tumor cells to conventional therapies. Nevertheless, ancillary biological effects remain largely overlooked in nanoparticle development, limiting the predictive value of current preclinical studies and restricting the translation of promising formulations into clinical applications [131,134].

Mechanism-driven design addresses these limitations through several complementary strategies. First, it requires a comprehensive characterization of nanoparticle transformations using advanced analytical techniques, including high-resolution electron microscopy (HR-TEM/STEM with EDXS and EELS), synchrotron-based X-ray spectroscopy (XANES/EXAFS), single-particle ICP-MS, and chromatographic methods [135,136,137]. Together, these techniques enable researchers to monitor structural, compositional, and oxidation-state changes under physiologically relevant conditions. Second, experimental models should reproduce the biochemical complexity of specific biological compartments rather than relying on simplified buffer systems that cannot capture competitive protein exchange or realistic ionic environments [129]. Third, the biological identity of nanoparticles, including potentially controllable nano–bio interactions discussed in Section 3.1, should be incorporated into mechanism-driven design rather than treated as an unpredictable consequence of systemic administration. Finally, integrating biotransformation kinetics into safety-by-design frameworks enables researchers to anticipate safety outcomes while exploiting predictable transformations for applications such as triggered drug release and multistage diagnostics [129].

Unlike conventional nanoparticle development, which optimizes physicochemical properties before evaluating biological performance, mechanism-driven design begins with the desired biological behavior. It considers how nanoparticles will transform in vivo, which biological responses they may induce, and which physiological barriers they must overcome [130,138]. Material properties are then engineered to support these biological objectives. By placing nano–bio interactions at the center of the design process, this framework offers a more predictive strategy for improving reproducibility and clinical translation [129,132].

### 7.2. Human-Relevant Preclinical Models

A major contributor to the translational crisis in nanomedicine is the limited predictive power of conventional preclinical models. Despite decades of advances in drug development, the vast majority of therapeutic candidates that successfully complete preclinical testing ultimately fail during clinical development because their efficacy or safety in humans cannot be accurately predicted [139]. High clinical attrition across pharmaceutical development more broadly illustrates the limited predictive capacity of conventional preclinical models, although these general drug-development statistics should not be interpreted as nanomedicine-specific failure rates [140,141,142]. This predictive gap is particularly relevant to nanomedicine because nanoparticle behavior is additionally influenced by complex and species-dependent nano–bio interactions.

This predictive gap is particularly pronounced for nanomedicines because their biological performance depends not only on the pharmacological activity of the therapeutic cargo but also on complex interactions between the nanoparticle and its biological environment. Following administration, nanoparticles undergo dynamic physicochemical transformations, acquire protein coronas, interact with immune cells, and experience transport processes that are strongly influenced by tissue architecture, vascular permeability, extracellular matrix composition, and biomechanical forces. Many of these processes are highly species-dependent and context-specific, making extrapolation from conventional in vitro and animal models particularly challenging [130,138,143].

Traditional two-dimensional (2D) cell cultures represent the simplest and most widely used preclinical platform, but fail to reproduce the structural and functional complexity of native tissues. Monocultures grown on flat substrates lack the three-dimensional organization, extracellular matrix, multicellular interactions, and physiological gradients that regulate nanoparticle transport, cellular uptake, and therapeutic response in vivo. Consequently, they frequently overestimate nanoparticle penetration and therapeutic efficacy while underrepresenting clinically relevant barriers to delivery [144,145,146].

Animal models partially overcome these limitations by providing intact physiological systems; however, their predictive value for human nanomedicine remains limited. Although orthotopic and genetically engineered disease models more closely resemble human pathology than conventional xenograft models, interspecies differences in anatomy, physiology, immune function, metabolism, and vascular biology continue to constrain their translational relevance. These differences influence nanoparticle biodistribution, protein corona composition, clearance mechanisms, and toxicity profiles, contributing to the poor concordance often observed between successful preclinical studies and subsequent clinical outcomes. In cancer nanomedicine, this limitation is particularly important because an animal model may reproduce antitumor activity while still providing an inaccurate prediction of human nanoparticle biodistribution, nano–bio interactions, tumor penetration, or the relationship between local drug exposure and therapeutic response. Moreover, animal experiments are costly, time-consuming, ethically constrained, and frequently unsuitable for high-throughput optimization of nanoparticle formulations [147,148].

Organ-on-a-chip (OoC) technology has emerged as one of the most promising approaches to address these shortcomings by recreating key aspects of human organ physiology within microengineered microfluidic platforms. Unlike conventional static cultures, OoC systems integrate three-dimensional tissue architecture with controlled fluid perfusion, mechanical stimulation, tissue–tissue interfaces, and physiologically relevant biochemical gradients. These features enable reconstruction of critical determinants of nanoparticle behavior that are largely absent from traditional in vitro models, including shear stress, cyclic mechanical strain, vascular transport, endothelial barrier function, and dynamic cell–cell communication [140,147,149].

Importantly, these physiological cues directly influence nanomedicine performance. Fluid shear stress regulates nanoparticle margination, endothelial adhesion, and cellular uptake, while cyclic mechanical forces alter epithelial permeability and inflammatory responses. For example, lung-on-a-chip models have demonstrated that breathing-induced mechanical deformation substantially modifies nanoparticle-induced toxicity, suggesting that conventional static cultures may underestimate pulmonary responses. Likewise, blood–brain barrier chips have shown that physiological flow conditions are essential determinants of nanoparticle translocation across the neurovascular interface, highlighting the importance of dynamic perfusion for accurately predicting central nervous system drug delivery. In oncology, tumor-on-a-chip platforms reproduce key features of the tumor microenvironment, including abnormal vascular permeability, extracellular matrix density, interstitial transport, and endothelial barriers, that collectively govern nanoparticle accumulation and penetration. These systems therefore provide a more physiologically relevant framework for evaluating nanomedicine delivery than either static cultures or simplified animal tumor models [150,151,152,153,154]. The predictive value of human-relevant models in cancer nanomedicine should therefore be assessed against specific translational endpoints rather than considered as a single global property. For biodistribution, relevant models should reproduce vascular transport, endothelial permeability, organ-specific sequestration, and clearance-related processes. For nano–bio interactions, they should capture exposure to human biological fluids, protein corona evolution, complement and immune-cell interactions, and patient-dependent biological variability. For tumor penetration, models should reproduce the combined effects of vascular architecture, extracellular matrix density, interstitial transport, and spatial tumor heterogeneity. Finally, prediction of therapeutic response requires integration of nanoparticle delivery with cellular uptake, drug release, target engagement, and tumor-cell sensitivity. No single current model reproduces all of these processes simultaneously; therefore, complementary model systems are likely to provide greater translational value than reliance on a single experimental platform.

The incorporation of human-derived primary cells, patient-derived organoids, and induced pluripotent stem cell (iPSC)-derived tissues further enhances the translational relevance of OoC platforms by reducing reliance on interspecies extrapolation [155]. Coupled with integrated biosensors capable of continuously monitoring barrier integrity, metabolic activity, oxygen consumption, inflammatory signaling, and other physiological parameters, these systems enable real-time assessment of dynamic biological responses that are difficult to capture using conventional endpoint assays. Such capabilities are particularly valuable for nanomedicines, whose interactions with biological systems evolve continuously throughout circulation and tissue exposure [147,156].

Beyond individual organs, multi-organ-on-a-chip and body-on-a-chip platforms represent an important step toward reproducing systemic human physiology. By interconnecting multiple tissue compartments through shared microfluidic circulation, these systems enable investigation of organ-organ communication, pharmacokinetics, metabolism, and secondary toxicities that cannot be captured using isolated tissue models. In experimental preclinical studies, integrated gastrointestinal–liver chips have demonstrated that nanoparticles administered through the intestinal compartment can induce downstream hepatic injury through inter-organ crosstalk, illustrating how systemic interactions influence nanomedicine safety. Such platforms offer the potential to evaluate absorption, distribution, metabolism, elimination, and toxicity within a single human-relevant experimental system [140,157,158].

Despite their considerable promise, important challenges remain before OoC technologies can be routinely incorporated into regulatory decision-making and pharmaceutical development pipelines. Device standardization, interlaboratory reproducibility, long-term tissue stability, scalability, manufacturing cost, and integration of fully functional immune and lymphatic systems continue to limit widespread implementation. Furthermore, although increasing evidence demonstrates improved physiological relevance, large-scale validation against clinical outcomes is still required to establish OoC platforms as qualified predictive tools for regulatory applications [147,159,160]. These findings demonstrate mechanistic and physiological advantages over conventional static models, but they should not yet be interpreted as evidence of validated clinical predictivity, as direct prospective correlation with human nanomedicine outcomes remains limited.

Overall, human-relevant preclinical models should be evaluated according to their ability to predict the specific biological processes that most strongly determine clinical nanomedicine performance. In cancer nanomedicine, these include systemic biodistribution, nano–bio interactions and immune recognition, tumor extravasation and penetration, cellular uptake, drug release, and therapeutic response. Organ-on-a-chip systems, patient-derived organoids, humanized models, and complementary advanced platforms may improve prediction of selected components of this cascade, but none currently provides a fully validated surrogate for human clinical outcomes. Their greatest translational value may therefore lie in their combined use to identify formulation-specific delivery barriers and biological variability before clinical development. Continued standardization and prospective validation against human pharmacokinetic, imaging, and therapeutic outcome data will be essential before these systems can be regarded as clinically predictive tools.

### 7.3. Rationalized Nanomaterial Standardization

Batch-to-batch (B2B) variability remains one of the most significant yet underappreciated barriers to the clinical translation of nanomedicines. Even when nominally identical synthesis protocols are employed, subtle variations in physicochemical properties, including particle size, surface chemistry, impurity profiles, colloidal stability, and protein corona formation, can substantially alter biological performance [78]. These inconsistencies are further exacerbated by the lack of harmonized characterization protocols and reporting standards across laboratories, limiting reproducibility and preventing meaningful comparison of studies [161,162].

The magnitude of this challenge has been demonstrated in one of the most comprehensive interlaboratory investigations conducted to date by Mülhopt et al., who synthesized and characterized 46 batches of five OECD priority nanomaterials across 14 European laboratories. Although particle size distributions were generally reproducible under standardized synthesis conditions, substantial batch-dependent differences were observed in trace elemental impurities and radical generation, with oxidative activity varying by as much as 24-fold despite otherwise similar physicochemical characteristics. These findings demonstrate that conventional characterization metrics alone are insufficient to ensure biological equivalence, particularly for nanomedicines whose interactions with biological systems are highly sensitive to nanoscale surface properties [78].

Such variability has important translational consequences. Small alterations in surface composition or contaminant levels may modify protein corona formation, cellular uptake, immune recognition, biodistribution, and therapeutic efficacy. For this reason, reproducible manufacturing should be regarded not merely as a quality control objective but as a prerequisite for establishing reliable structure-activity relationships and achieving predictable clinical performance [128,163,164].

However, variability introduced during synthesis represents only one component of the broader reproducibility crisis. Sharifi et al. highlight that standardized characterization methodologies remain inconsistently applied throughout the academic nanomedicine community, while analytical verification of commercially sourced nanomaterials is frequently omitted despite well-documented differences between suppliers and production batches. As a result, studies often investigate materials whose true physicochemical characteristics differ substantially from those reported, compromising the reproducibility and comparability of published findings [78,90].

The implications extend beyond individual experiments. Mahmoudi has argued that limited transparency in experimental reporting contributes to the broader reproducibility crisis affecting nanomedicine, where methodological details, unsuccessful experiments, and negative findings are frequently underreported. Similarly, Leong et al. emphasize that many reported nano-bio interactions are valid only within narrowly defined experimental conditions. Improving reproducibility, therefore, requires not only more comprehensive reporting but also explicit documentation of the experimental design space, enabling readers to evaluate the robustness and generalizability of published observations [78,165].

Addressing these challenges requires coordinated standardization across synthesis, characterization, reporting, and regulatory evaluation. One important initiative is the Minimum Information Reporting in Bio-Nano Experimental Literature (MIRIBEL) framework, which establishes minimum reporting requirements for material characterization, biological assays, and experimental protocols. Adoption of MIRIBEL has the potential to substantially improve transparency and facilitate cross-study comparisons. Nevertheless, several authors have emphasized that reporting requirements should remain adaptable, as characterization strategies must ultimately reflect the intended biological application and the specific physicochemical properties of each nanomaterial platform [165].

Complementing reporting standards, international organizations including ISO Technical Committee 229, the European Nanomedicine Characterization Laboratory (EUNCL), and the US National Cancer Institute Nanotechnology Characterization Laboratory (NCI-NCL) have developed standardized operating procedures covering physicochemical characterization, in vitro testing, and in vivo evaluation. These efforts represent an essential step toward harmonized quality assessment, although their widespread adoption by academic laboratories and journal reviewers remains inconsistent [166,167,168,169].

Metrological infrastructure also requires further development. Certified reference materials (CRMs) and reference test materials (RTMs) provide traceable benchmarks for validating analytical methods, ensuring measurement comparability, and supporting regulatory decision-making. However, as highlighted by Abram et al., currently available nanoscale reference materials remain largely restricted to particle size measurements, with comparatively few standards available for surface chemistry, particle concentration, colloidal stability, or biologically relevant dispersion media. Moreover, the limited long-term stability of many nanoparticle reference dispersions complicates their use throughout extended preclinical development programmes [170].

Future progress will therefore depend on integrating standardized manufacturing with harmonized analytical characterization and transparent reporting practices. Routine verification using complementary orthogonal characterization techniques, interlaboratory validation studies, wider implementation of certified reference materials, and publication of negative or inconsistent findings should become standard practice rather than exceptions. Together, these measures would transform nanomedicine research from a collection of isolated observations into a robust and reproducible evidence base capable of supporting reliable regulatory evaluation and successful clinical translation [166,171].

### 7.4. Scalable and GMP-Compatible Manufacturing

The transition from bench-scale synthesis to industrial Good Manufacturing Practice (GMP)-compliant manufacturing remains one of the principal barriers to the clinical translation of nanomedicines [172]. Although laboratory-scale protocols routinely generate proof-of-concept nanomaterials, translating these processes into robust, large-scale manufacturing is considerably more challenging [173]. This “lab-to-factory gap” arises because relatively few studies systematically connect quality target product profile (QTPP)-defined clinical requirements with the engineering principles governing nanoparticle production, scalable process design, and regulatory control strategies. Consequently, manufacturing processes that perform reliably at laboratory scale often fail to maintain product consistency under industrial conditions [172,174,175].

A central challenge is the preservation of predefined critical quality attributes (CQAs), including physicochemical characteristics, drug loading, encapsulation efficiency, sterility, and release behavior, across increasing production scales. Even modest changes in particle size distribution, surface properties, or morphology can significantly alter biodistribution, therapeutic efficacy, immunogenicity, and safety [176]. As production volumes increase, maintaining batch-to-batch reproducibility becomes progressively more difficult owing to changes in mixing dynamics, mass and heat transfer, residence time distributions, and downstream processing. These challenges are particularly pronounced for formulations containing sensitive therapeutic payloads, such as nucleic acids, which require stringent process conditions to preserve structural integrity throughout manufacturing [177,178].

Addressing these challenges requires manufacturing strategies that integrate pharmaceutical quality principles with engineering design. Quality by Design (QbD) has therefore become the dominant framework for nanomedicine development because it explicitly links critical material attributes (CMAs) and critical process parameters (CPPs) to product CQAs, enabling science-based process understanding and control. Early definition of the QTPP and associated CQAs facilitates rational formulation development, establishes clinically relevant acceptance criteria, and guides process optimization throughout the product lifecycle [177,179]. Combined with Process Analytical Technology (PAT), design-of-experiments (DoE), multivariate data analysis, and edge-of-failure mapping, QbD enables real-time process monitoring, risk-based control strategies, and continued process verification. Since the introduction of the FDA’s Process Analytical Technology initiative, regulatory expectations have increasingly shifted toward real-time quality assurance rather than reliance on end-product testing alone [172,179,180]. This paradigm is particularly important for nanomedicines and other non-biological complex drugs (NBCDs), for which product “sameness” cannot be established using a single structural descriptor but instead depends on comprehensive physicochemical characterization together with well-controlled manufacturing processes. Consequently, chemistry, manufacturing, and controls (CMC) strategies are expected to evolve throughout clinical development, progressing from risk-based process understanding during early development to fully validated manufacturing processes and comprehensive control strategies before commercialization [172,181].

Advances in manufacturing technologies have substantially improved the scalability of nanoparticle production. Microfluidic and millifluidic platforms enable rapid and highly controlled mixing, producing nanoparticles with narrow size distributions while preserving key CQAs during scale-up. Recent developments in parallelized hydrodynamic flow-focusing devices and confined impinging jet mixers have increased production throughput to industrially relevant levels without compromising formulation quality. Commercial platforms such as NanoAssemblr have further facilitated translation by providing standardized manufacturing systems that span discovery, preclinical, and clinical production [21,176,182]. Across multiple studies, lipid- and polymer-based nanoparticles produced by microfluidic or impingement mixing consistently maintain particle sizes within the desired therapeutic range when critical engineering parameters, including Reynolds number and mixing time, are preserved. Continuous manufacturing offers additional advantages by reducing batch-to-batch variability, enabling flexible production volumes without process redesign, and facilitating integration with PAT-based control systems. Furthermore, closed single-use manufacturing platforms reduce contamination risks, simplify facility operation, and improve GMP compliance. Nevertheless, challenges remain, including channel fouling, clogging, equipment scalability, and the need for specialized manufacturing infrastructure, although advances in millifluidic designs and more durable fabrication materials are progressively mitigating these limitations [172,178,183].

Successful industrial translation ultimately depends on controlling the entire chemistry, manufacturing, and controls (CMC) workflow rather than individual unit operations alone. Raw material quality, excipient selection, solvent removal, impurity control, facility segregation, and downstream purification all influence the reproducibility and regulatory acceptability of the final product. Manufacturing processes involving multiple synthetic steps, low-yield reactions, or highly sensitive operating conditions frequently compromise robustness and increase the risk of batch-to-batch or site-to-site variability [173,178,184]. These challenges are particularly evident for PLGA-based nanoparticles, where no universally applicable manufacturing process exists, and individual formulations require product-specific process development and validation [177]. Comprehensive orthogonal analytical characterization, including dynamic light scattering, asymmetric flow field-flow fractionation coupled with multi-angle light scattering, and residual solvent analysis, is therefore essential for batch release, comparability assessment, and lifecycle management [172].

The successful commercialization of nanomedicines such as Doxil/CAELYX, Onpattro, Comirnaty, Abraxane, and AmBisome demonstrates that clinical translation depends less on formulation novelty than on robust process understanding, scalable manufacturing technologies, and scientifically justified control strategies. These examples illustrate that integration of QbD, PAT, orthogonal analytical characterization, and GMP-compliant manufacturing provides the foundation for reproducible large-scale production. Future progress will therefore depend not only on advances in nanoparticle design but also on the continued development of standardized, data-driven, and regulatory-aligned manufacturing platforms capable of delivering consistent product quality throughout the entire product lifecycle [172,185,186].

### 7.5. Regulatory Science Integration

The regulatory landscape for nanomedicines remains fragmented, uncertain, and poorly aligned with the complexity of nano-enabled therapeutics, making regulatory approval one of the major barriers to clinical translation. Although research on nanomedicine has expanded rapidly, comparatively little attention has been devoted to developing harmonized regulatory frameworks, standardized safety assessment, and long-term toxicity evaluation [187,188]. Existing regulatory pathways were largely established for conventional small-molecule drugs and biologics, yet nanomedicines exhibit unique physicochemical properties, pharmacokinetic profiles, and biodistribution patterns that cannot be predicted from their individual components. Consequently, nanoparticles may accumulate in specific organs, alter local drug exposure, and generate toxicities that would not be anticipated from the active pharmaceutical ingredient (API) alone. In addition, the multicomponent composition of nanomedicines, including carriers, stabilizers, and excipients, introduces further safety concerns because each component may contribute to unexpected biological responses [143,178,181].

A further challenge arises from inconsistent regulatory classification. Definitions of nanotechnology-based medicinal products remain non-uniform across jurisdictions, resulting in different regulatory pathways for similar products. This issue is particularly evident for borderline products, whose classification depends on their intended clinical application. For example, iron oxide nanoparticles may be regulated as medicinal products when used for diagnostic imaging but as medical devices when employed as surgical aids. Such inconsistencies complicate product development, increase regulatory uncertainty, and delay global commercialization [187,189,190].

Beyond classification, existing safety evaluation frameworks remain inadequate for nano-specific hazards [179]. Regulatory agencies generally assess nanomedicines on a case-by-case basis using testing strategies adapted from conventional pharmaceuticals. However, these approaches often fail to capture critical nano-specific characteristics, including altered biodistribution, bioaccumulation, surface reactivity, protein corona formation, and interactions with the immune system [128,179]. Because nanoparticles are predominantly cleared by phagocytic immune cells, immunotoxicity represents a central safety concern that cannot be fully addressed using conventional toxicological paradigms. Moreover, animal models routinely employed for small-molecule drugs frequently show limited predictive value for human immune responses, while immunodeficient xenograft models used in oncology provide little insight into nanoparticle immunogenicity. Although current regulatory requirements include comprehensive toxicological testing, including acute and repeat-dose toxicity, safety pharmacology, genotoxicity, developmental toxicity, immunotoxicity, and carcinogenicity, these studies were not designed to evaluate the distinctive biological behavior of nanoscale materials [191,192].

Methodological limitations further complicate regulatory evaluation. Many standardized analytical methods currently used for pharmaceutical development were originally established for soluble chemicals and cannot be directly applied to nanomedicines. Their performance often depends on nanoparticle composition, size, and surface chemistry, requiring validation across representative nanomaterial classes such as liposomes, polymeric nanoparticles, and iron oxides. The REFINE (Regulatory Science Framework for Nano(bio)material-based Medical Products and Devices) project has highlighted several important methodological gaps, including the absence of standardized methods for quantifying large therapeutic payloads such as nucleic acids, limited applicability of existing assays for endotoxin detection and protein corona characterization, and insufficient validation of analytical methods across diverse nanomaterial platforms [181,193,194]. To address these challenges, REFINE has developed physiologically based pharmacokinetic (PBPK) models and decision-support tools to guide nanomedicine characterization and regulatory testing [185]. Similarly, the OECD Working Party on Manufactured Nanomaterials has made substantial progress in adapting existing Test Guidelines for nanomaterials, although further refinement is required to account for agglomeration behaviour, surface transformations, and dynamic interactions within biological environments [136,187,195].

Recognizing these limitations, regulatory science is increasingly shifting towards proactive, science-based approaches. The Safe-by-Design (SbD) framework incorporates safety considerations during the earliest stages of nanomedicine development by optimizing physicochemical properties, exposure scenarios, and lifecycle characteristics before clinical translation. This strategy aims to improve biodistribution, minimize off-target accumulation, and enhance long-term biocompatibility without compromising therapeutic efficacy [187,196,197,198]. Complementing these efforts, research infrastructures such as the Nanomedicine Characterization Laboratory (NCL) and the European Nanomedicine Characterization Laboratory (EUNCL) have established standardized analytical workflows and extensive reference datasets to support preclinical characterization and regulatory evaluation [181,199].

Collectively, these initiatives demonstrate that the principal challenge is no longer simply the absence of scientific knowledge but the lack of internationally harmonized implementation. Although agencies such as the FDA and the European Medicines Agency have introduced nano-specific initiatives, comprehensive and legally binding regulatory guidance remains limited [190]. Future progress will therefore depend on harmonized physicochemical characterization protocols, validated nano-specific testing methods, greater integration of academic and regulatory data, and internationally coordinated regulatory frameworks. Such advances will reduce approval uncertainty, facilitate cross-jurisdictional acceptance of safety data, and accelerate the clinical translation of safe and effective nanomedicines [200].

### 7.6. Adaptive and Biomarker-Guided Clinical Trial Design

Interpatient variability in nanoparticle biodistribution, pharmacokinetics, tumor microenvironment characteristics, receptor expression, and immune status may contribute to heterogeneous treatment responses and dilution of therapeutic effects in conventional clinical trials [164,201,202]. Adaptive and biomarker-guided trial designs could therefore be particularly relevant to cancer nanomedicine by enabling prospective enrichment of patient populations according to molecular, imaging, or clinical features associated with nanoparticle delivery or therapeutic response. Such approaches may allow preferential enrollment of patients most likely to benefit, early discontinuation of ineffective treatment arms, and more efficient evaluation of dose or formulation variants [203,204,205,206,207,208]. However, direct clinical evidence supporting adaptive enrichment specifically in nanomedicine remains limited, and these approaches should currently be regarded as promising translational strategies rather than established practice.

Clinical experience with adaptive designs derives primarily from precision oncology rather than nanomedicine. Trials such as I-SPY 2 and BATTLE have demonstrated the feasibility of using biomarker-defined subgroups, Bayesian decision rules, and adaptive treatment allocation to identify populations more likely to benefit from investigational therapies [209,210]. Importantly, these trials did not directly evaluate nanomedicine formulations and therefore provide proof-of-concept rather than direct evidence for nanomedicine-specific benefit. Methodological approaches such as subgroup-based Bayesian adaptive enrichment (SUBA) and adaptive seamless designs could in future be applied to nanomedicine trials using validated imaging, molecular, or pharmacokinetic biomarkers to guide patient selection, dose optimization, or formulation selection [211,212,213].

Important limitations must nevertheless be recognized. Adaptive enrichment requires reliable predictive biomarkers and sufficiently rapid clinical or surrogate endpoints, while repeated interim analyses require rigorous statistical control of multiplicity and type I error [211,214,215,216]. Operational complexity, biomarker validation, and real-time data requirements may further limit implementation. Consequently, adaptive trial designs should presently be considered an emerging strategy for cancer nanomedicine. Prospective studies directly evaluating whether biomarker-guided adaptive approaches improve the clinical development of nanomedicines will be required before their translational value can be established.

## 8. Conclusions

Cancer nanomedicine has emerged as one of the most promising approaches for improving the delivery, efficacy, and safety of anticancer therapeutics. Over the past three decades, advances in nanoparticle engineering have generated a vast body of preclinical evidence demonstrating enhanced tumor accumulation, controlled drug release, and improved therapeutic indices. Nevertheless, the translation of these successes into clinical practice has been considerably more limited than initially anticipated. This discrepancy constitutes the translational paradox of cancer nanomedicine: while technological innovation continues to accelerate, clinical adoption and patient benefit have progressed at a much slower pace.

The factors underlying this paradox are multifaceted and interconnected. Biological barriers, including tumor heterogeneity, variable vascular permeability, and the inconsistent manifestation of the enhanced permeability and retention (EPR) effect in human tumors, challenge the predictive value of preclinical findings. At the same time, limitations in animal models, insufficient standardization of nanoparticle characterization, manufacturing complexities, regulatory uncertainties, and economic constraints further hinder clinical translation. Many nanomedicine platforms that demonstrate exceptional performance in laboratory settings fail to reproduce comparable outcomes in patients.

**Figure 5:**
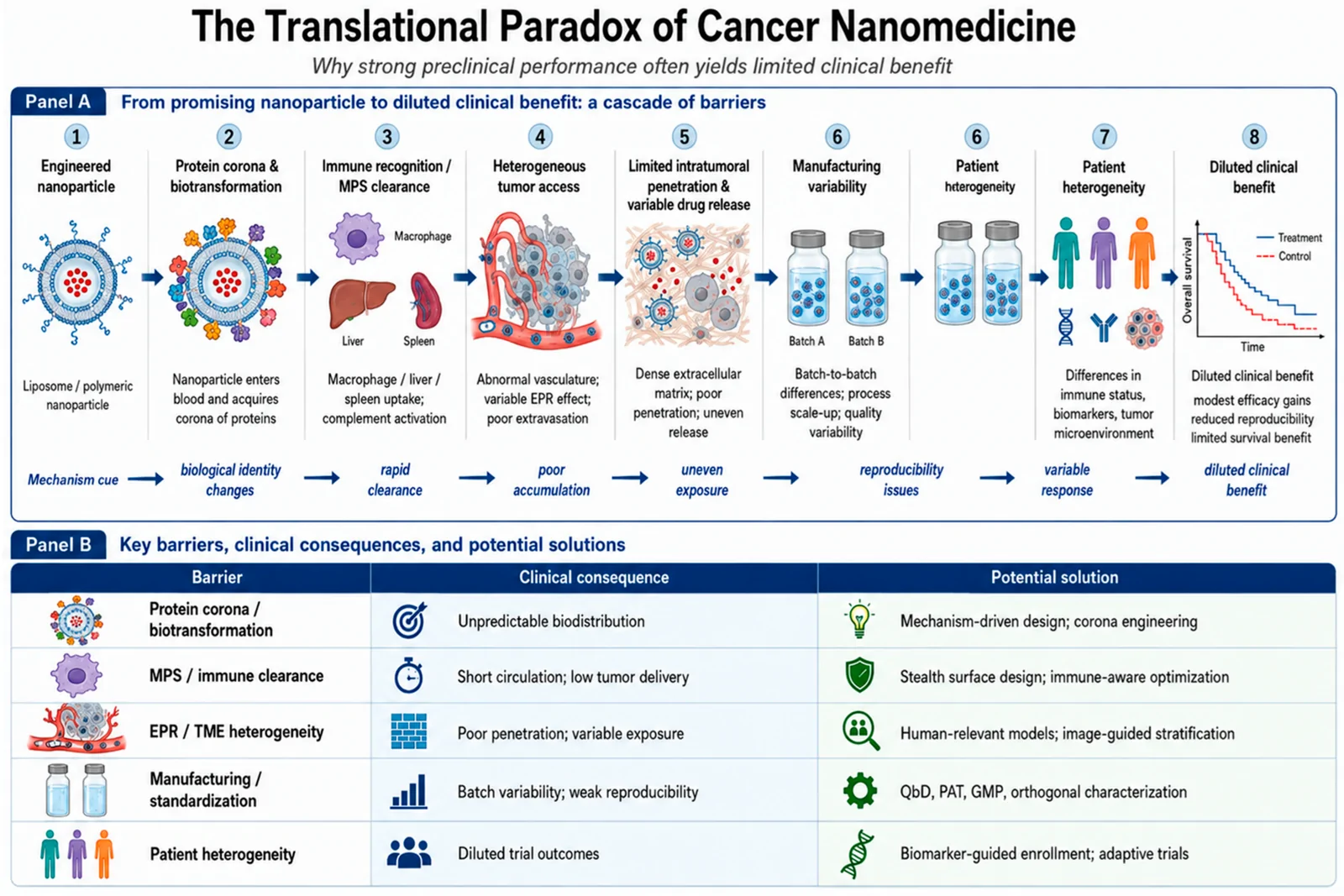
Interconnected biological, pharmacokinetic, manufacturing, and patient-related barriers contributing to the translational paradox of cancer nanomedicine, together with selected strategies proposed to improve clinical translation.

The interconnected barriers contributing to translational failure and the corresponding strategies proposed to improve clinical translation are summarized in Figure 5. On the basis of the evidence discussed throughout this review, several priorities can be identified for future cancer nanomedicine development. First, improving the predictability of nano–bio interactions should be considered a fundamental priority, because protein corona formation, immune recognition, clearance, biodistribution, and biological transformation ultimately determine whether the physicochemical properties optimized during formulation development are preserved in vivo. Mechanism-driven design should therefore increasingly incorporate biological identity and transformation from the earliest stages of nanocarrier development.

Second, preclinical evaluation should move toward complementary human-relevant models that are validated against clinically relevant endpoints, including biodistribution, vascular transport, tumor penetration, cellular uptake, toxicity, and therapeutic response. Organoids, organ-on-a-chip systems, human-derived tissues, and appropriately selected animal models should be regarded as complementary rather than interchangeable tools, with their predictive value assessed against specific components of the nanomedicine delivery cascade. Third, standardized physicochemical and biological characterization should be implemented early and consistently, because reproducibility of material properties and experimental conditions is a prerequisite for meaningful comparison between studies and for reliable regulatory evaluation.

Fourth, scalability and manufacturing robustness should be treated as design requirements rather than late-stage development problems. GMP-compatible processes, Quality by Design, Process Analytical Technology, validated critical quality attributes, and reproducible batch-to-batch manufacturing should therefore be integrated before extensive clinical investment. These approaches represent among the most mature and immediately actionable strategies for reducing translational failure. Fifth, clinical development should increasingly incorporate biomarker-guided patient selection, particularly when nanoparticle accumulation, target expression, or tumor microenvironment characteristics are expected to influence treatment response. Adaptive or enrichment-based trial designs may eventually complement this strategy, although their specific value in nanomedicine still requires prospective clinical validation.

Collectively, these priorities suggest that future progress will depend less on increasing nanoparticle complexity and more on improving predictability across the entire translational pathway. The most immediate gains are likely to arise from rigorous characterization, reproducible manufacturing, and early regulatory integration, while improved biological predictability, validated human-relevant models, and biomarker-guided clinical development represent essential areas for continued translational research. Aligning these elements from formulation design through clinical evaluation may help transform cancer nanomedicine from a predominantly technology-driven field into a more predictive, reproducible, and patient-oriented therapeutic discipline.

## Figures and Tables

**Table 1:** Examples of clinically approved nanomedicine formulations used in oncology.

Formulation	Active Substance	Type of Nanocarrier	Indication	Status	Source
Doxil/Caelyx	Doxorubicin hydrochloride	PEGylated liposome	Ovarian cancer, AIDS-related Kaposi Sarcoma, multiple myeloma	FDA approved in 1995, EMA 1996	[92,93]
Pazenir	Paclitaxel	Albumin-bound nanoparticle	Metastatic breast cancer, metastatic pancreatic adenocarcinoma, NSCLC	Approved in EU (2019)	[94]
Abraxane	Paclitaxel	Protein-bound particles	Breast cancer, NSCLC, metastatic adenocarcinoma of the pancreas	FDA approved in 2005, EMA 2008	[95,96]
Onivyde	Irinotecan	PEGylated liposome	Metastatic pancreatic adenocarcinoma, administered in combination with oxaliplatin, fluorouracil, and leucovorin as first-line therapy, or with fluorouracil and leucovorin following progression after gemcitabine-based therapy.	FDA approved in October 2015, label expansion on 13 February 2024;	[97,98]
Vyxeos (CPX-351)	Daunorubicin + cytarabine	Liposomal co-formulation	Acute myeloid leukemia (AML)	FDA approved on 3 August 2017, EMA 2018	[99,100]
Nanoxel	Docetaxel	Polymeric micelle	Breast cancer and other solid tumors	Approved in India	[101]
Myocet	Doxorubicin	Liposomes	Metastatic breast cancer	Approved in EU (2000)	[102]
Mepact	Mifamurtide	Liposomes	Non-metastatic resectable osteosarcoma	Approved in EU (2009)	[103]

Abbreviations: AIDS, acquired immunodeficiency syndrome; AML, acute myeloid leukemia; EMA, European Medicines Agency; EU, European Union; FDA, U.S. Food and Drug Administration; NSCLC, non-small-cell lung cancer; PEG, polyethylene glycol.

**Table 2:** Examples of investigational, discontinued, terminated, or withdrawn nanomedicine formulations in oncology and their principal translational outcomes.

Formulation	Active Substance	Type of Nanocarrier	Indication	Status	Reason for Failure/Discontinuation or Current Development Status	Source
ThermoDox	Doxorubicin	Thermosensitive liposomes	Pancreatic cancer, liver tumors	Phase III completed; clinical development discontinued	Insufficient efficacy/futility. Phase III OPTIMA was completed, but interim analysis indicated futility and the sponsor subsequently discontinued further survival follow-up and did not pursue the program toward regulatory approval.	[104,105]
Marqibo	Vincristine sulfate	Liposome	Acute lymphoblastic leukemia (ALL)	Accelerated FDA approval (2012); approval withdrawn (2022)	Voluntary regulatory withdrawal following accelerated approval; withdrawal requested by the sponsor.	[106]
DaunoXome	Daunorubicin citrate	Liposome	Advanced HIV-associated Kaposi sarcoma	FDA approved (1996); later discontinued commercially	Commercial discontinuation; not classified as clinical or pharmacological failure.	[107,108]
DepoCyt	Cytarabine	Sustained-release liposome (DepoFoam)	Lymphomatous meningitis (neoplastic meningitis)	Previously approved; EU marketing authorization withdrawn in 2018	Commercial discontinuation. Marketing authorization was withdrawn at the holder’s request following permanent discontinuation of marketing for commercial reasons.	[109]
MM-302	Doxorubicin	HER2-targeted immunoliposome	HER2-positive metastatic breast cancer	Development discontinued after Phase II (HERMIONE trial)	Insufficient efficacy/futility. Phase II HERMIONE was terminated after DMC review and futility analysis indicated no benefit over control.	[110,111]
MM-310	Docetaxel	EphA2-targeted liposome	Advanced solid tumors, pancreatic cancer	Clinical development terminated (Phase I)	Safety/unfavorable therapeutic index. Development discontinued after emerging cumulative Grade 3 peripheral neuropathy and review of Phase I safety data indicated that an optimal therapeutic index was unlikely to be achieved.	[112]
BIND-014	Docetaxel	PSMA-targeted polymeric nanoparticle	Advanced solid tumors, NSCLC, prostate cancer	Clinical development discontinued (Phase II)	Strategic/financial discontinuation rather than a uniform efficacy failure. Development across indications was not continued; individual cohorts were stopped for differing reasons, including enrollment and portfolio considerations, and the sponsor subsequently entered Chapter 11 proceedings.	[113]
MRX34	miR-34a mimic	Liposomal nanoparticle	Advanced solid tumors, hepatocellular carcinoma	Phase I terminated because of severe immune-related adverse events	Safety/toxicity. Phase I terminated following five immune-related serious adverse events.	[114,115]
CALAA-01	siRNA (targeting RRM2)	Cyclodextrin-based polymer nanoparticle	Advanced solid tumors	Phase I completed; development discontinued	Clinical development terminated; specific reason not stated in the primary trial registry.	[116,117]
CRLX101	Camptothecin	Cyclodextrin-containing polymer nanoparticle	Ovarian cancer, renal cell carcinoma, NSCLC, other solid tumors	Phase I/II completed; development discontinued	Insufficient efficacy/futility. Later sponsor-led Phase II development was terminated following a futility decision, although other investigator/NCI studies were completed.	[118,119]
SPI-077	Cisplatin	Stealth PEGylated liposome	Ovarian cancer, solid tumors	Phase II completed; development discontinued due to limited efficacy	Insufficient efficacy. Phase II studies showed limited objective clinical activity despite favorable tolerability.	[120,121]
LE-SN38	SN-38	Liposome	Metastatic colorectal cancer	Phase II completed; not approved	Phase II completed; development not advanced to approval. Reason for subsequent discontinuation not specified in the primary registry.	[122,123]
NK105	Paclitaxel	Polymeric micelle	Breast cancer, gastric cancer, pancreatic cancer, solid tumors	Phase III completed; not approved	Failure to meet the primary endpoint. The Phase III trial failed to demonstrate prespecified non-inferiority versus paclitaxel for progression-free survival.	[124,125]
NanoTherm	Iron oxide	Iron oxide NP	Glioblastoma	Previously CE-marked in Europe for the treatment of brain tumors; prior CE marking subsequently discontinued; currently investigational.	Ongoing clinical development. Currently being evaluated in the recruiting ANCHIALE study in recurrent glioblastoma.	[126]

Abbreviations: ALL, acute lymphoblastic leukemia; CE, Conformité Européenne; DMC, Data Monitoring Committee; EphA2, ephrin type-A receptor 2; EU, European Union; FDA, U.S. Food and Drug Administration; HER2, human epidermal growth factor receptor 2; HIV, human immunodeficiency virus; miR-34a, microRNA-34a; NCI, National Cancer Institute; NP, nanoparticle; NSCLC, non-small-cell lung cancer; PSMA, prostate-specific membrane antigen; RRM2, ribonucleotide reductase regulatory subunit M2; siRNA, small interfering RNA.

**Table 3:** Relative maturity of strategies proposed to improve the clinical translation of cancer nanomedicine.

Maturity Category	Strategy	Current Translational Role
Established/implementation-ready	Standardized physicochemical characterization and reporting	Already central to reproducibility, quality control, and regulatory assessment
Established/implementation-ready	GMP-compliant manufacturing	Required for clinical and commercial production
Established/implementation-ready	Quality by Design (QbD) and Process Analytical Technology (PAT)	Established pharmaceutical frameworks for process understanding, control, and product quality
Established/implementation-ready	Early regulatory and CMC integration	Established development principle for complex pharmaceutical products
Emerging	Human-relevant models, including organ-on-a-chip and patient-derived systems	Increasingly useful for selected translational endpoints, but clinical predictive validity remains incompletely established
Emerging	Mechanism-driven nanomedicine design	Growing framework for incorporating nano–bio interactions and biological transformation into formulation design
Emerging	Biomarker-guided patient selection	Clinically established in precision oncology, but still developing specifically for nanomedicine
Emerging	Adaptive clinical trial designs for nanomedicine	Established methodology in oncology more broadly, but limited direct application to nanomedicine
Conceptual/future-oriented	Rational protein-corona engineering	Promising experimental strategy with limited clinical validation
Conceptual/future-oriented	AI-assisted nanomedicine-specific patient stratification	Potential future tool; direct clinical validation in nanomedicine remains limited

**Abbreviations:** AI, artificial intelligence; CMC, chemistry, manufacturing, and controls; GMP, Good Manufacturing Practice; PAT, Process Analytical Technology; QbD, Quality by Design.

## Data Availability

No new data were created or analyzed in this study. Data sharing is not applicable to this article.
